# Cardiometabolic disease risk markers are increased following burn injury in children

**DOI:** 10.3389/fpubh.2023.1105163

**Published:** 2023-06-02

**Authors:** Sofina Begum, Samantha Lodge, Drew Hall, Blair Z. Johnson, Sze How Bong, Luke Whiley, Nicola Gray, Vanessa S. Fear, Mark W. Fear, Elaine Holmes, Fiona M. Wood, Jeremy K. Nicholson

**Affiliations:** ^1^Harvard Medical School, Harvard University, Boston, MA, United States; ^2^Channing Division of Network Medicine, Brigham and Women’s Hospital, Boston, MA, United States; ^3^Department of Metabolism, Digestion and Reproduction, Faculty of Medicine, Imperial College London, London, United Kingdom; ^4^Australian National Phenome Centre, Computational and Systems Medicine, Health Futures Institute, Perth, WA, Australia; ^5^School of Biomedical Sciences, The University of Western Australia, Perth, WA, Australia; ^6^Perron Institute for Neurological and Translational Science, Nedlands, WA, Australia; ^7^Centre for Computational and Systems Medicine, Health Futures Institute, Murdoch University, Perth, WA, Australia; ^8^Translational Genetics, Telethon Kids Institute, Perth, WA, Australia; ^9^WA Department of Health, Burns Service of Western Australia, Perth, WA, Australia; ^10^Faculty of Medicine, Institute of Global Health Innovation, London, United Kingdom

**Keywords:** metabolomics, precision medicine, cardiometabolic disease risk, child health, burn injury, NMR, inflammation, cytokines

## Abstract

**Introduction:**

Burn injury in children causes prolonged systemic effects on physiology and metabolism leading to increased morbidity and mortality, yet much remains undefined regarding the metabolic trajectory towards specific health outcomes.

**Methods:**

A multi-platform strategy was implemented to evaluate the long-term immuno-metabolic consequences of burn injury combining metabolite, lipoprotein, and cytokine panels. Plasma samples from 36 children aged 4–8 years were collected 3 years after a burn injury together with 21 samples from non-injured age and sex matched controls. Three different ^1^H Nuclear Magnetic Resonance spectroscopic experiments were applied to capture information on plasma low molecular weight metabolites, lipoproteins, and α-1-acid glycoprotein.

**Results:**

Burn injury was characterized by underlying signatures of hyperglycaemia, hypermetabolism and inflammation, suggesting disruption of multiple pathways relating to glycolysis, tricarboxylic acid cycle, amino acid metabolism and the urea cycle. In addition, very low-density lipoprotein sub-components were significantly reduced in participants with burn injury whereas small-dense low density lipoprotein particles were significantly elevated in the burn injured patient plasma compared to uninjured controls, potentially indicative of modified cardiometabolic risk after a burn. Weighted-node Metabolite Correlation Network Analysis was restricted to the significantly differential features (q <0.05) between the children with and without burn injury and demonstrated a striking disparity in the number of statistical correlations between cytokines, lipoproteins, and small molecular metabolites in the injured groups, with increased correlations between these groups.

**Discussion:**

These findings suggest a ‘metabolic memory’ of burn defined by a signature of interlinked and perturbed immune and metabolic function. Burn injury is associated with a series of adverse metabolic changes that persist chronically and are independent of burn severity and this study demonstrates increased risk of cardiovascular disease in the long-term. These findings highlight a crucial need for improved longer term monitoring of cardiometabolic health in a vulnerable population of children that have undergone burn injury.

## 1. Introduction

Non-severe burn injury, defined as less than 20% total body surface area (TBSA), is a common childhood medical insult, accounting for over 85% of pediatric burn cases in Australia (1). Recent research has demonstrated that even non-severe childhood burns are associated with a significant increase in hospitalization for a range of morbidities, including cardiovascular diseases, suggestive of a sustained systemic response to this type of injury (2). However, non-severe burn injury has traditionally been less well-studied than severe burns (2). Severe burn injury induces a global multisystemic effect, far beyond the initial site of injury, and the subsequent hypermetabolic response has been shown to persist for several years post-injury, even when no scarring remains (3). However, the extent and nature of this response in non-severe burns is less well established.

The burn-induced hypermetabolic response is thought to result from a physiological drive toward healing, which causes elevated catabolism (4), inflammation and sustained stress hormone release (5). The hypermetabolic state has been found to be persistent in many patients with severe burns, with its resolution linked to the longevity and duration of exposure to elevated levels of catecholamines, cortisol and glucagons (6). Serum cortisol measurements, and a range of cytokines including IL-6 and TNF-α have been reported to remain significantly increased for up to 3 years post-burn injury in children (7). This heightened proinflammatory state is typically accompanied by hyperglycemia and increased insulin resistance, independent of burn severity (8). Post-burn dysregulation of metabolism and immune regulation has also been associated with increased production of reactive oxygen species (ROS) and reactive nitrogen species such as nitric oxide and peroxynitrite (9). While severe burns have been well documented to induce this chronic inflammatory and hypermetabolic response, the mechanisms behind these chronic aberrations and whether these changes persist after non-severe burns, is less clear (10, 11).

One of the hallmarks of burn injury is the high rate of hospital admissions for cardiovascular disease (CVD) compared to the general population within the first few years post-injury (12). However, some researchers report no such elevation in CVD risk (13). Our prior work, deploying targeted metabolic phenotyping strategies to map the hypermetabolic state present in children long after recovery from a non-severe burn injury, demonstrated increased plasma levels of multiple amino acids and the neurotoxic metabolite quinolinic acid (14) but did not assess whether there are sustained metabolic changes that might be associated with the observed increased risk of cardiovascular disease (15). In order to address this substantial research gap, here we utilize ^1^H Nuclear Magnetic Resonance (NMR) spectroscopy to measure a wide panel of plasma metabolites. We apply three different pulse sequences to capture different molecular panels including lipoproteins (quantified based on the standard 1D NMR pulse sequence); small molecules (spin echo pulse sequence to attenuate contributions of macromolecules) and the JEDI pulse sequence (combining three spectroscopic editing techniques: diffusion; relaxation; J-editing to suppress small molecules and large proteins/lipoproteins) to capture biomarkers of inflammation. Together these pulse sequences enabled assessment of the metabolic landscape associated with the risk and incidence of cardiovascular disease (16). We identify a systemic signature of dysregulated lipoproteins associated with prior burn injury that may be linked to altered CVD risk post burn-injury.

## 2. Results

### 2.1. Metabolic phenotypes indicate persistent metabolic effects of burn injury in children

**Figure 1:**
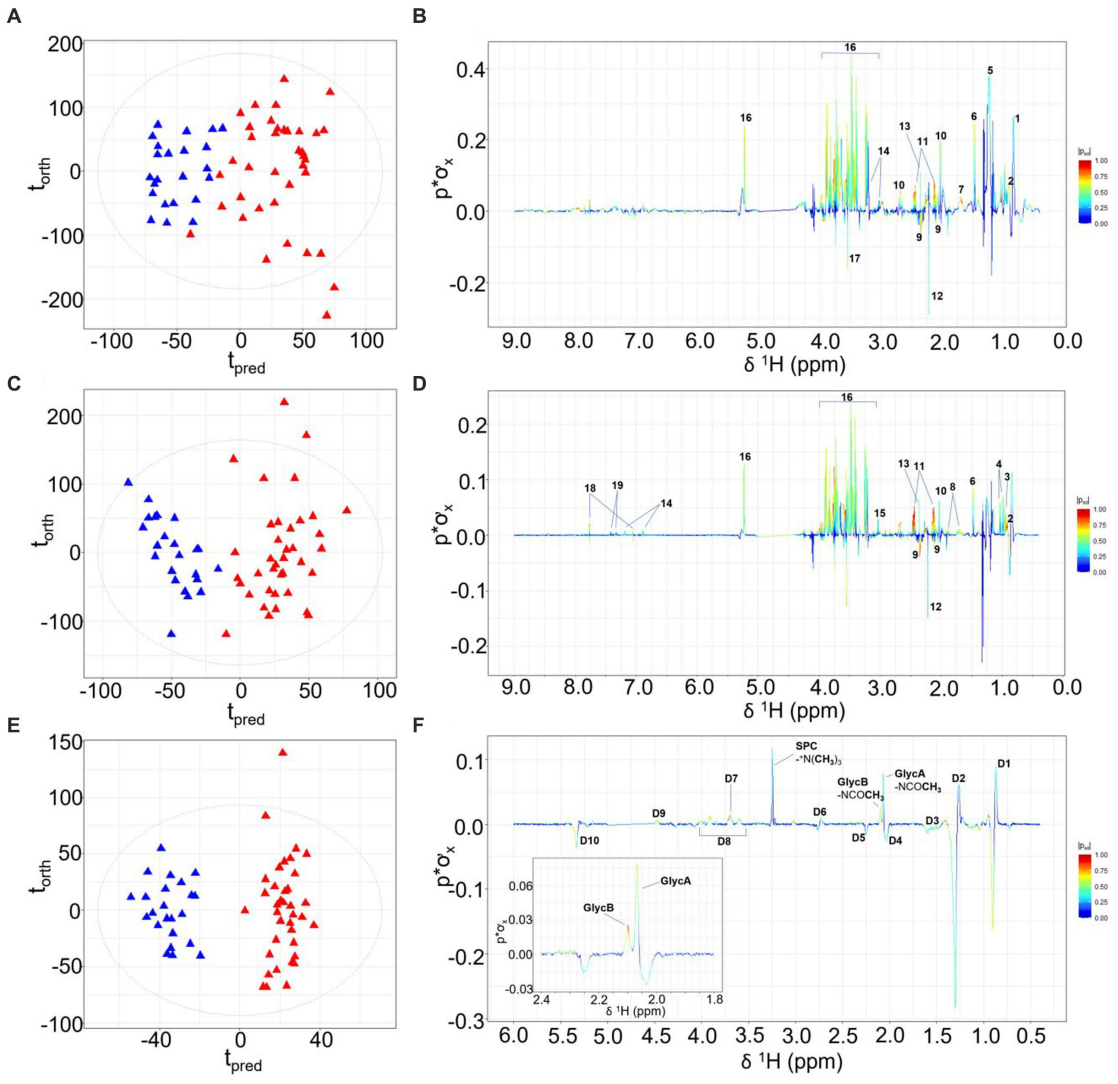
OPLS-DA scores plots, with t_orth_ (orthogonal) representing the greatest amount of systematic variation in the data set uncorrelated to Y (burn injury or healthy control groups), and t_pred_ (predictive) representing the greatest amount of correlated variation to Y. Corresponding model loadings coefficients are shown, differentiating the NMR plasma spectra from children with prior burn injury (red) from the control group (blue) based on the standard 1D NMR pulse sequence **(A,B)**; the CPMG pulse sequence **(C,D)** and the JEDI-PGPE pulse sequence **(E,F)**. Key: 1- Lipid: -CH_2_CH_3_, 2-Isoleucine, 3-Leucine, 4-Valine, 5- Lipid: -CH_2_CH_2_CH_2_–6-Alanine, 7- Lipid: -CH_2_CH_2_CO 8-Lysine 9-Glutamate, 10-Methioine, 11-Glutamine, 12-Acetone, 13-Pyruvate, 14-Tyrosine, 15-Creatine, 16-Glucose, 17-Glycine, 18-3-methylhistidine, 19-Phenylalanine, D1-Lipid: -CH_2_CH_3_ D2- Lipid: -CH_2_CH_2_CH_2_- D3- Lipid: -CH_2_CH_2_CO D4- Lipid: -CH_2_CH=CH- D5- Lipid: -CH_2_CO D6- Lipid: -CH=CH-CH_2_-CH=CH- D7- SPC: ^+^NCH_2_CH_2_OP- D8-GlycA/GlycB *N*-acetyl sugar moieties, D9- SPC:-^+^NCH_2_CH_2_OP- D10- Lipid: -CH=CH-.

Differentiation of children with prior burn injury versus no burn injury was apparent for all three data sets focusing on lipoproteins, small molecules, and the inflammatory NMR panel (17). Orthogonal Partial Least Squares-Discriminant Analysis (OPLS-DA) is a robust statistical strategy to interrogate features in high-dimensional data, utilizing supervised regression analysis to define variation for class discrimination (18). The OPLS-DA models of all three sets of spectral data indicated there were significant metabolic differences between the prior burn injury and the control groups. The strongest classifier of burn injury was based on the standard 1D NMR pulse sequence dataset suggesting that burn injury was defined by a broad metabolic response as the standard profile contains contributions from low molecular weight molecules, lipoproteins, and glycoproteins. The cross-validated OPLS-DA model yielded a predictive value (Q^2^Y) of 0.52 and was predominantly driven by higher relative concentrations of glucose, amino acids (with the exception of glutamate and glycine), pyruvate, creatine, *N*-acetyl signals from α-1-acid glycoproteins (GlycA and GlycB) representing the burn injury group, with lower concentrations of acetate, acetoacetate, glutamate, and 3-methyl histidine (Figures 1A,B). The contribution of these low molecular weight metabolites differentiating the prior burn injury group from controls was further assessed using metabolite concentrations quantified using B.I.-LISA™ software (19). Glutamine, alanine, creatine, phenylalanine, and glucose were found to be directly correlated with burn injury, based on Student’s t test, while controlling the false-discovery rate (*p* < 0.05) and the Cliff’s delta value (Supplementary Figure S2B), whereas glutamic acid and 3-D-hydroxybutyric acid were inversely associated (Table 1). 3-D-hydroxybutyric acid was higher in the control group plasma samples but did not retain significance after controlling the false discovery rate (Figures 1C,D). There was a trend toward higher levels of other ketone bodies (acetone and acetoacetate) in the control group, but these were not statistically significant.

**Table 1:** Quantified metabolites differentiating children with prior burn injury from control.

Compound	Mean concentration [SD] Control group	Mean concentration [SD] Burn injury group	Cliff’s Delta^a^	value of *p* (univariate)	*q*-value^b^
Glutamine: glutamate ratio	2.190 [2.762]	5.565 [3.595]	0.56	0.0003***	0.004**
Glutamine	0.503 [0.090]	0.638 [0.100]	0.53	5.30×10^−3^***	5.88×10^−3^***
Alanine	0.278 [0.058]	0.348 [0.095]	0.48	0.0017**	0.013*
GlycA^c^	4.386×10^3^	4.518 × 10^3^	0.45	0.037*	0.104
Creatine	0.046 [0.017]	0.054 [0.009]	0.37	0.017*	0.097
Phenylalanine	0.034 [0.043]	0.039 [0.020]	0.35	0.022*	0.098
Glucose	4.607 [0.962]	4.966 [1.179]	0.34	0.027*	0.106
GlycB^c^	0.986×10^3^ [0.110×10^3^]	1.087 × 10^3^ [0.174 × 10^3^]	0.31	2.78×10^−4^***	0.016*
3-D- Hydroxybutyric acid	0.086 [0.278]	0.061 [0.299]	−0.30	0.047*	0.150
Glutamate	0.213 [0.108]	0.101 [0.077]	−0.55	3.80×10^−3^***	5.88×10^−3^***

a Ranked in order of the magnitude of the Cliff’s Delta statistic.

b Value of *p* corrected for multiple testing (FDR, BH), *p < 0.05, **p < 0.01, ***p < 0.001.

c GlycA (α-1-acid glycoprotein signal A) and GlycB (α-1-acid glycoprotein signal B) are imputed integrals of spectral peaks.

Whereas the standard 1-Dimensional (1D) NMR experiment reflects the global molecular signature and composition, the Carr-Purcell Meiboom-Gill (CPMG) spin-echo experiment, effects spectral editing based on the proton T_2_ relaxation properties of proton signals by attenuating peaks from large molecules with slow rotational correlation times such as lipoproteins (20). In contrast JEDI-PGPE pulse sequences use pulsed field gradients to enhance signals from molecules with high segmental motional freedom but slow translational diffusion molecules that are contained within certain lipoprotein sub-compartments (21). Since the OPLS-DA model constructed from the standard 1D NMR spectra (Q^2^Y = 0.52) was stronger than either the CPMG (Q^2^Y 0.46; Figures 1C,D) or JEDI-PGPE (Q^2^Y 0.26; Figures 1E,F) models, this suggests that a broad range of low molecular weight molecules (most prevalent through 1D NMR spectral resolution), lipoproteins and glycoproteins are disrupted in children who have suffered prior burn injury. The CPMG model loadings (Figure 1D) further highlighted the association of citrate and formate with thermal injury whereas GlycA and GlycB were the strongest discriminatory signals differentiating burn from control group in the JEDI-PGPE data set (Figure 1F). The signals arising from the supramolecular phospholipid composite (SPC) peak were reduced in the burn injury group, but were not significantly differential. The corresponding PCA scores plots are provided in Supplementary Figure S1.

Metabolite-metabolite correlations were explored for key metabolites characteristic of prior burn injury using STOCSY (22) (Supplementary Figure S3). To investigate the association between 3-D-hydroxybutyric acid and ketogenesis, a correlation analysis driven from the apex of the acetoacetate peak (Supplementary Figure S3A) demonstrated a strong association between the three ketone bodies and 3-D-hydroxybutyric acid, further suggesting that burn injury was associated with a systematic decrease in plasma ketone bodies. Glucose was directly associated with GlycA and HDL lipoproteins (Supplementary Figure S3B); pyruvate was weakly associated with alanine and lactate (Supplementary Figure S3C); creatine was positively associated with multiple amino acids (Supplementary Figure S3D) and glutamine and glutamate were strongly inversely correlated (Supplementary Figure S3E).

### 2.2. Prior burn injury affects the plasma lipoprotein composition

**Figure 2:**
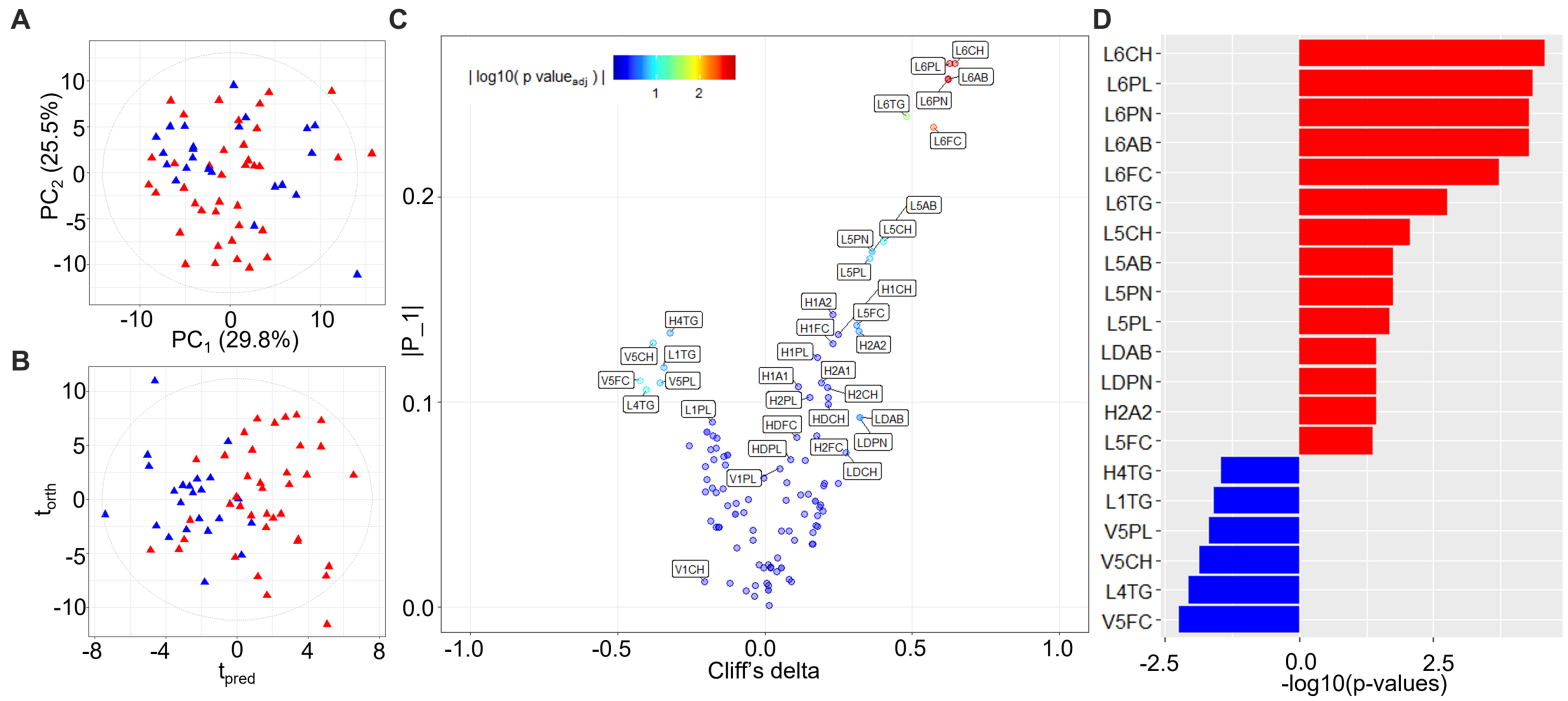
PCA and OPLS-DA models of quantified lipoprotein subfractions. **(A)** PCA scores plot of burn injury patients and healthy control profiles (R^2^Y 0.987, Q^2^Y 0.933); **(B)** Scores plot of the equivalent OPLS-DA model (R^2^X 0.437, R^2^Y 0.492, Q^2^Y 0.321); **(C)** Variable importance eruption plot of predictive lipoprotein parameters based on OPLS-DA component loadings (t_pred_) and univariate effect sizes (Cliff’s delta). Co-ordinates are colored by the FDR-adjusted value of *p* (<0.05) from the statistical group comparison; **(D)** Significant lipoprotein parameters ranked by Cliff’s delta. Significant lipoprotein parameters distinguishing burn injury source (scald vs. flame) from multivariate linear regression modeling can be found in Supplementary Figure S4. L6CH, Low-Density Lipoprotein- 6 Subclass Cholesterols; L6PL, Low-Density Lipoprotein- 6 Subclass Phospholipids; L6PN, Low-Density Lipoprotein- 6 Subclass Particle Number; L6AB, Low-Density Lipoprotein- 6 Subclass Apolipoprotein-B100; L6FC, Low-Density Lipoprotein- 6 Subclass Free Cholesterol; L6TG, Low-Density Lipoprotein- 6 Subclass Triglycerides; L5CH, Low-Density Lipoprotein- 5 Subclass Cholesterols; L5AB, Low-Density Lipoprotein- 5 Subclass Apolipoprotein-B100; L5PN, Low-Density Lipoprotein- 5 Subclass Particle Number; L5PL, Low-Density Lipoprotein- 5 Subclass Phospholipids; LDPN, Low-Density Lipoprotein- Particle Number; LDAB, Low-Density Lipoprotein- Apolipoprotein-B100; H2A2, High-Density Lipoprotein- 2 Subclass Apolipoprotein-A2; L5FC, Low-Density Lipoprotein- 5 Subclass Free Cholesterol; H4TG, High-Density Lipoprotein- 4 Subclass Triglycerides; L1TG, Low-Density Lipoprotein- 1 Subclass Triglycerides; V5PL, Very Low-Density Lipoprotein- 5 Subclass Phospholipids; V5CH, Very Low-Density Lipoprotein- 5 Subclass Cholesterols; L4TG, Low-Density Lipoprotein- 4 Subclass Triglycerides; V5FC, Very Low-Density Lipoprotein- 5 Subclass Free Cholesterols.

PCA modeling of lipoproteins, quantified according to the Bruker iVDR B.I.-LISA™ method (19), showed a cluster of burn injury patients in the lower left-hand quadrant of the scores plot (Figure 2A). The differential signature of the plasma lipoprotein parameters was further defined in the OPLS-DA model (R^2^X 0.437, R^2^Y 0.492, Q^2^Y 0.321) and the eruption plot based on the model loadings versus Cliff’s delta statistic (Figures 2B,D). Prior burn injury was characterized by relatively higher plasma concentrations of LDL subfractions 5 and 6, particularly particle number, total cholesterol, free cholesterol, triglycerides, and Apolipoprotein B. Total LDPN and LDAB were also elevated in the burn injury group although this association was not significant after adjusting for multiple testing. In contrast, multiple VLDL5 subfractions were present in lower concentrations in the prior burn injury group in comparison to the controls as were L1TG and L4TG (Figures 2C,D; Table 2). Although the group size was small, the prior burn injury group was subdivided into burns caused by scalds versus flame. LDL-1 and LDL-4 triglyceride fractions were associated with scalds whereas the L6PL subfraction was associated with flame burns (Supplementary Figure S4).

**Table 2:** Quantified plasma lipoproteins significantly differentiating children with prior burn injury from healthy controls (*p* < 0.05).

Lipoprotein subfraction^a^	Mean concentration [SD] control	Mean concentration [SD] Burn injury	Cliff’s Delta^b^	*p*-value	*q*-value^c^
L6CH	11.86 [3.39]	17.61 [4.24]	0.65	1.10×10^−5^***	1.50×10^−3^***
L6PL	7.46 [1.69]	10.16 [2.21]	0.63	4.60×10^−5^***	1.50×10^−3^***
L6PN	174.52 [46.07]	248.11 [60.73]	0.62	2.50×10^−5^***	1.50×10^−3^***
L6AB	9.60 [2.53]	13.65 [3.34]	0.62	5.50×10^−5^***	1.50×10^−3^***
L6FC	3.80 [0.94]	5.14 [1.09]	0.57	2.00×10^−4^***	4.33×10^−3^***
L6TG	2.50 [0.58]	3.15 [0.92]	0.48	1.80×10^−3^***	3.31×10^−2^***
L5CH	7.66 [2.34]	10.41 [2.58]	0.4	8.80×10^−2^***	0.11
L5AB	5.37 [1.56]	7.06 [1.69]	0.36	0.018*	0.17
L5PN	97.70 [28.44]	128.34 [30.78]	0.36	0.018*	0.17
L5PL	4.72 [1.16]	5.92 [1.29]	0.35	0.021*	0.17
LDPN	790.10 [181.46]	935.38 [167.27]	0.32	0.036*	0.22
LDAB	43.45 [9.98]	51.45 [9.20]	0.32	0.036*	0.22
H2A2	2.89 [0.63]	3.15 [0.87]	0.32	0.038*	0.22
L5FC	3.01 [0.63]	3.60 [0.65]	0.31	0.043*	0.24
H4TG	3.02 [0.60]	2.75 [0.78]	−0.32	0.036*	0.22
L1TG	3.83 [1.34]	3.20 [1.28]	−0.34	0.026*	0.19
V5PL	1.26 [0.34]	1.07 [0.50]	−0.36	0.021*	0.17
V5CH	0.84 [0.34]	0.63 [0.48]	−0.38	0.014*	0.15
L4TG	1.33 [0.42]	1.07 [0.57]	−0.4	9.00×10^−3^***	0.11
V5FC	0.64 [0.23]	0.41 [0.26]	−0.42	5.90×10^−3^***	0.09

a L6CH, Low-Density Lipoprotein- 6 Subclass Cholesterols; L6PL, Low-Density Lipoprotein- 6 Subclass Phospholipids; L6PN, Low-Density Lipoprotein- 6 Subclass Particle Number; L6AB, Low-Density Lipoprotein- 6 Subclass Apolipoprotein-B100; L6FC, Low-Density Lipoprotein- 6 Subclass Free Cholesterol; L6TG, Low-Density Lipoprotein- 6 Subclass Triglycerides; L5CH, Low-Density Lipoprotein- 5 Subclass Cholesterols; L5AB, Low-Density Lipoprotein- 5 Subclass Apolipoprotein-B100; L5PN, Low-Density Lipoprotein- 5 Subclass Particle Number; L5PL, Low-Density Lipoprotein- 5 Subclass Phospholipids; LDPN, Low-Density Lipoprotein- Particle Number; LDAB, Low-Density Lipoprotein- Apolipoprotein-B100; H2A2, High-Density Lipoprotein- 2 Subclass Apolipoprotein-A2; L5FC, Low-Density Lipoprotein- 5 Subclass Free Cholesterol; H4TG, High-Density Lipoprotein- 4 Subclass Triglycerides; L1TG, Low-Density Lipoprotein- 1 Subclass Triglycerides; V5PL, Very Low-Density Lipoprotein- 5 Subclass Phospholipids; V5CH, Very Low-Density Lipoprotein- 5 Subclass Cholesterols; L4TG, Low-Density Lipoprotein- 4 Subclass Triglycerides; V5FC, Very Low-Density Lipoprotein- 5 Subclass Free Cholesterols.

b *p*-value corrected for multiple testing (FDR, BH), *p < 0.05, **p < 0.01, ***p < 0.001.

c Ranked in order of the magnitude of the Cliff’s Delta statistic.

### 2.3. Correlation of and other inflammatory markers lipoproteins with cytokines

**Figure 3:**
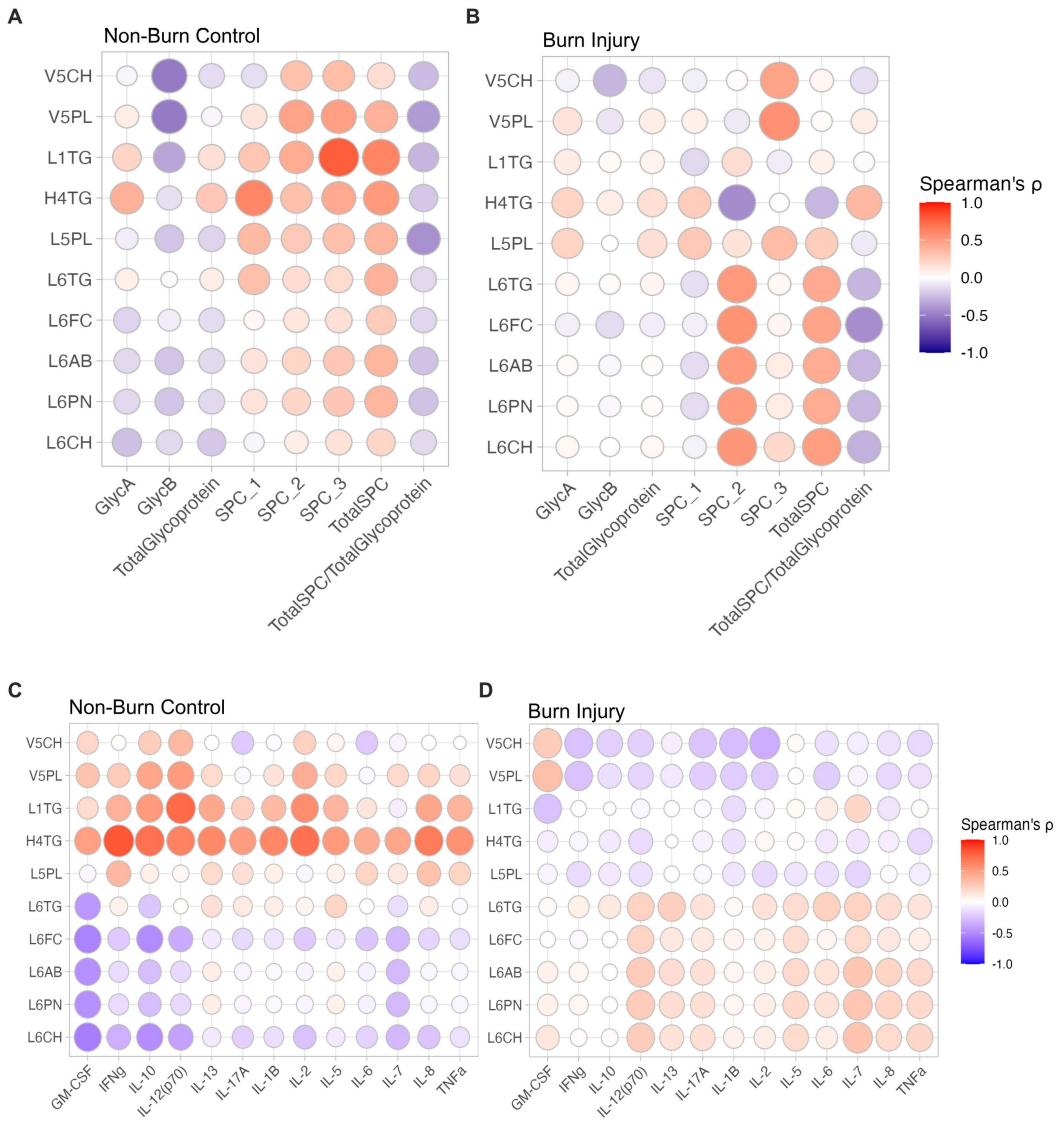
Spearman’s correlation between lipoproteins and JEDI-PGPE quantified integrals for **(A)** non-burn controls and **(B)** burn injury patients; and between cytokines and differential lipoproteins **(C)** non-burn controls and **(D)** burn injury patients. GlycA, α-1-acid glycoprotein signal A; GlycB, α-1-acid glycoprotein signal B; SPC1, Supramolecular Phospholipid Composite Integral 1; SPC2, Supramolecular Phospholipid Composite Integral 2; SPC3, Supramolecular Phospholipid Composite Integral 3; TotalSPC, Total Supramolecular Phospholipid Composite; GM-CSF, Granulocyte-macrophage colony-stimulating factor; IFNγ, Interferon γ; IL-10, Interleukin 10, IL-12(p70), Interleukin 12 heterodimeric 70kDA; IL-13, Interleukin 13; IL-17A, Interleukin 17A; IL-1B, Interleukin 1B; IL-2, Interleukin 2; IL-5, Interleukin 5; IL-6, Interleukin 6; IL-7, Interleukin 7; IL-8, Interleukin 8; TNF-α, Tumour Necrosis Factor-α.

**Figure 4:**
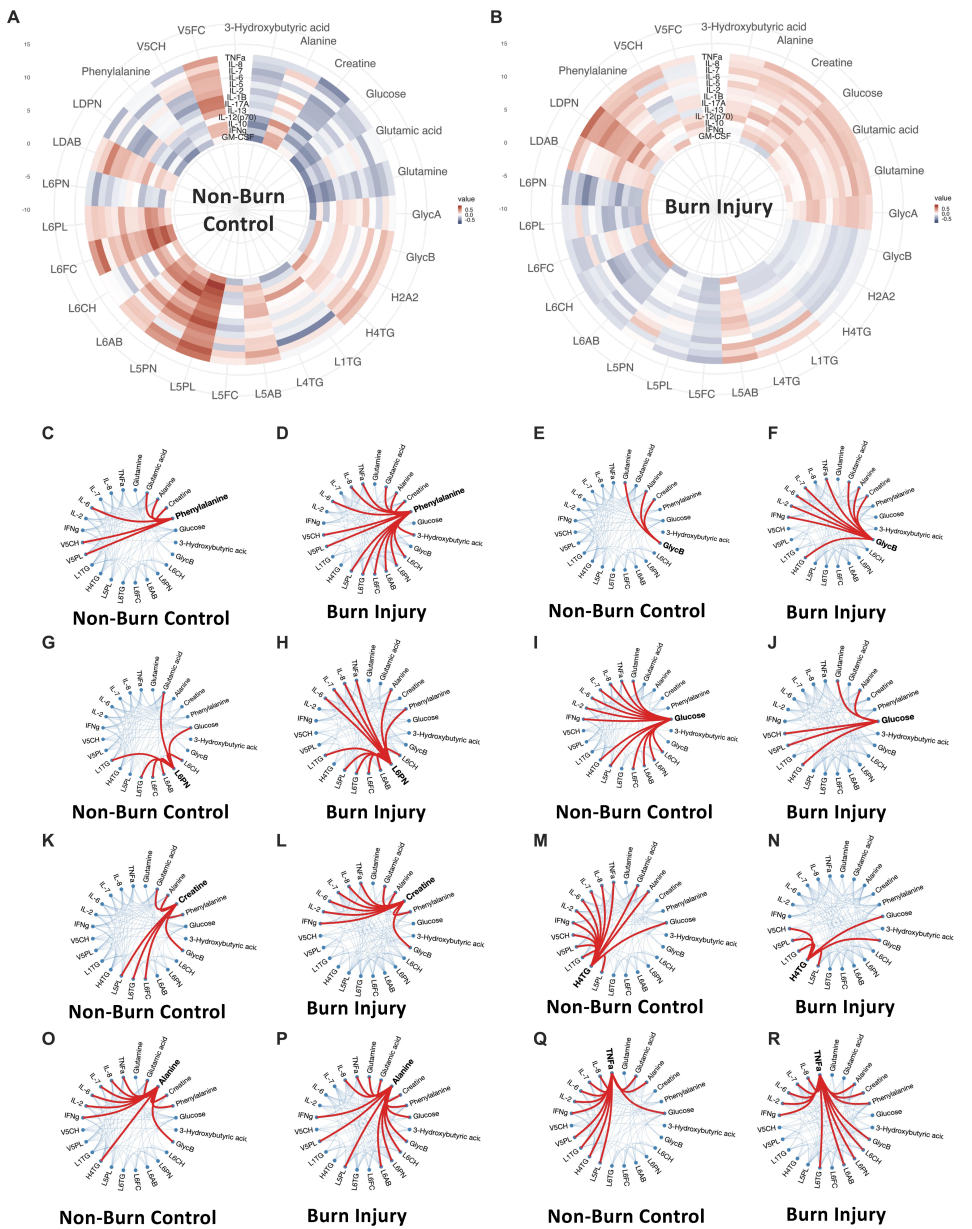
**(A,B)** Circular correlation map for healthy control **(A)** and prior burn injury **(B)** groups. **(C–R)** Weighted-node correlation networks showing inter-connectedness of significant parameters (*p* < 0.05) from the integrated three panels (cytokine, metabolites and lipoprotein) for control and burn injury groups highlighting key nodes. Additionally, IL-6 and IL-8 are included in the correlation maps based on their importance following burn injury. L6CH, Low-Density Lipoprotein- 6 Subclass Cholesterols; L6PL, Low-Density Lipoprotein- 6 Subclass Phospholipids; L6PN, Low-Density Lipoprotein- 6 Subclass Particle Number; L6AB, Low-Density Lipoprotein- 6 Subclass Apolipoprotein-B100; L6FC, Low-Density Lipoprotein- 6 Subclass Free Cholesterol; L6TG, Low-Density Lipoprotein- 6 Subclass Triglycerides; L5CH, Low-Density Lipoprotein- 5 Subclass Cholesterols; L5AB, Low-Density Lipoprotein- 5 Subclass Apolipoprotein-B100; L5PN, Low-Density Lipoprotein- 5 Subclass Particle Number; L5PL, Low-Density Lipoprotein- 5 Subclass Phospholipids; LDPN, Low-Density Lipoprotein- Particle Number; LDAB, Low-Density Lipoprotein- Apolipoprotein-B100; H2A2, High-Density Lipoprotein- 2 Subclass Apolipoprotein-A2; L5FC, Low-Density Lipoprotein- 5 Subclass Free Cholesterol; H4TG, High-Density Lipoprotein- 4 Subclass Triglycerides; L1TG, Low-Density Lipoprotein- 1 Subclass Triglycerides; V5PL, Very Low-Density Lipoprotein- 5 Subclass Phospholipids; V5CH, Very Low-Density Lipoprotein- 5 Subclass Cholesterols; L4TG, Low-Density Lipoprotein- 4 Subclass Triglycerides; V5FC, Very Low-Density Lipoprotein- 5 Subclass Free Cholesterols; GlycA, α-1-acid glycoprotein signal A; GlycB, α-1-acid glycoprotein signal B; IFNγ, Interferon γ; IL-2, Interleukin 2; IL-6, Interleukin 6; IL-7, Interleukin 7; IL-8, Interleukin 8; TNFα, Tumor Necrosis Factor α.

α-1-acid glycoprotein *N*-acetyl signals (GlycA and GlycB) have long been known to be associated with systemic inflammation (23), Spearman’s correlation of lipoprotein parameters and JEDI-PGPE integrals for GlycA, GlycB and SPC were calculated and presented two distinct profiles corresponding to non-burn controls (Figure 3A) and burn injury (Figure 3B). In the control group there were negative correlations between GlycA and IL-7, IL-10 and GM-CSF (Figure 4A), whereas GlycB was positively correlated with several cytokines including IFN-γ, IL-13, IL-17a, IL-13, IL-6 and TNF-α, signatures absent in burn injury children(Figure 4B). In contrast, in the burn injury group both GlycA and GlycB showed strong direct correlations for IL-2, IL-6, IL-10, IL-13, TNF-α and IFN-γ reflecting the hyperinflammatory consequences associated with previous burn injury (7). Of these cytokines, IL-2, TNF-α and IFN-γ showed independent association with burn injury.

The same contrasting correlation profile for the control and prior burn (Figures 3C,D) groups was also evident in the correlation structure between the significant lipoprotein parameters and the cytokines. The cytokine correlation map for the full list of lipoproteins is provided in Supplementary Figure S5. The small dense LDL lipoprotein parameters from subclasses 5 and 6 were weakly anticorrelated with most measured cytokines in the control group, particularly GM-CSF, IL-7, IL-10, and IL-12, whereas the VLDL subfraction 5 parameters (V5PL, V5CH, V5FC) were weakly directly associated with most cytokines, particularly IL-2, IL-10 and IL-12. This pattern largely reversed in the prior burn injury group with a general positive correlation between cytokines and LDL-6 lipoprotein parameters with notable correlations with IL-7 and IL-12. VLDL5 sub-particles were inversely related with the global cytokine profile for the burn group. Plasma HDTG concentrations were lower in the burn group. Interestingly, H2TG, H3TG, H3A1 and H3A2 were positively correlated to GlycA in burn injury but showed no correlation in non-burn signatures.

The global correlation structure in these data can be visualized as circular correlation maps, illustrating Spearman’s correlations for the panels of metabolites, lipoproteins and cytokines that significantly differentiate the prior burn participants from the control group (Figure 4). Additionally, IL-6 and IL-8 were included in the network based on their previous reported sustained association with burn injury (7). A contrasting correlation pattern between cytokines and metabolites/lipoproteins was observed when the control group was compared to the prior burn injury group. Whereas the overall correlation between individual cytokines and metabolites/lipoproteins was slightly weaker in the prior burn injury group due to the presence of sub-phenotypes within this group (Supplementary Figure S6), the number of connections between nodes for the correlation networks was substantially greater for most metabolites and cytokines in the burn injury group, indicating a co-ordinated immune-driven systemic response to burn injury. The notable exceptions were H4TG and glucose, which demonstrated a greater number of connections to cytokines in the control group. Phenylalanine was the node with the greatest number of weighted correlations (*n* = 15), linked with LDL and VLDL parameters, TNF-α, IL-6, IL-8, glutamate, alanine, creatine and 3-D-hydroxybutyrate in the prior burn injury group, whereas for the control group, there was a lower number of significant associations (*n* = 6), losing the association with the LDL parameters, TNF-α and IL-8 correlation, despite a cluster of strong immuno-metabolic connections presented in the circular correlation map (Figure 4A). GlycB correlated with 9 parameters, including 3 cytokines in the burn injury group, while in the control group, GlycB only mapped to glutamine, alanine and creatine (all elevated following burn injury). Similarly, the small dense lipoprotein parameters (e.g., L6PN) had higher weighted node correlations with cytokines and alanine and phenylalanine in the burn group.

## 3. Discussion

### 3.1. Immuno-inflammatory signatures of burn injury

We identified an inflammatory signature in the plasma profile of children with prior burn injury based on elevated concentrations of GlycB. This is consistent with our previous observation of elevated cytokines TNF-α (1.31-fold), IL-2 (1.18-fold), IL-7 (1.63-fold), and IFN-γ indicating that children with prior burns manifested low grade chronic inflammation. GlycA, and GlycB, represent glycosylated amino sugars in sidechains of a composite of five main acute phase glycoproteins: α-1-acid glycoprotein, α-1-antichymotrypsin, α-1-antitrypsin, haptoglobin and transferrin. GlycA and GlycB are robust markers of inflammation, found to be superior in accuracy to traditional pathology measures such as C-Reactive Protein (CRP) (24). Elevations in these *N*-acetylated glycoproteins have been reported in association with non-ischemic heart failure (25), vascular aging (26), rheumatoid arthritis (27), SARS-CoV-2 infection (21), insulin resistance (28) and atherosclerosis (29). Glycosylation patterns of serum proteins are typically expected during acute phase injury. Connelly et al., found in children suffering acute Kawasaki disease, elevated GlycA, increased total and small LDL particle numbers and decreased HDL particle numbers (*p* < 0.001) (30). Similar signatures were observed in this cohort of children, with elevated GlycA and GlycB and elevated small dense LDL particle numbers (mainly L6PN). GlycB also demonstrated significant correlations with TNF-α, IFN-γ, IL-2, IL-6, IL-7 and IL-8 exclusively in burn injury children, suggesting that it is associated with the systemic response to inflammation post burn injury (due to overlap with lipoprotein peaks, the higher concentrations of GlycA in the prior burn injury group did not reach statistical significance). Burn-induced increase in TNF-α concentrations can increase the rate of lipolysis and hence production of free fatty acids (31), which can contribute to the hyperglycemia and increased insulin resistance associated with post burn injury (32).

Johnston et al., demonstrated elevated levels of TNF-α (1.31-fold), IL-2 (1.18-fold), IL-7 (1.63-fold), and IFN-γ (1.18-fold) 3 years post injury in the pediatric burn injury patients in comparison to healthy controls and described a significantly reduced antibody response to diphtheria, tetanus, and pertussis vaccine antigens (7). Numerous studies have reported high levels of serum cytokines and chemokines post burn injury including IL-7, IL-10, IL-12, macrophage inflammatory protein-1b with some studies noting the sustained elevation of these cytokines for at least 3 years post injury (33). Sustained elevation of cytokines such as TNF-α, IL-2 and IL-17 at 3 years post burn may suggest a transition from a Th1 response in acute burn injury toward a Th2 dominated immune response in the chronic phase (6).

### 3.2. The metabolic ‘memory’ of prior burn injury is associated with distinctive lipoprotein signatures

Multiple plasma lipoproteins were systematically altered in participants with prior burn injury. Specifically, we found higher concentrations of small dense LDL subfractions (LDL5 and LDL6) to be associated with burn injury. These small dense LDL particles are known to have greater atherogenic potential than larger LDL subfractions (LDL1-4) and in particular, small dense LDL cholesterol is more predictive of cardiovascular disease than that of total LDL-C (34). A large population-based study also found that increased long-term risk of ischemic heart disease in men was related to preferential accumulation of small dense LDL particles but not large LDL particles (35). This raises the question as to whether the association of LDL5 and LDL6 cholesterol and particle number could have implications with respect to increased risk of cardiovascular disease following burn injury. In one study, higher LDL particle number was consistently associated with increased risk for cardiovascular disease, independent of other lipid measurements, but other LDL parameters were not associated with cardiovascular disease after adjustment for cholesterol concentrations (36). There is substantial evidence demonstrating associations between burn injury and cardiometabolic diseases (37). Hypertriglyceridemia, hypocholesterolemia, and hypophospholipidemia have been reported to occur following thermal injury (38) with excessive accumulation of triacylglycerols in liver tissue (39). Concentrations of both low- and high-density lipoprotein have been reported to be reduced following burn injury, which is consistent with depleted blood cholesterol and phospholipid levels that occur following burn injury (40). While the altered lipid profiles may certainly contribute the increased risk of cardiometabolic disease, the systemic effect of burn injury is complex and it is likely that immune dysfunction and inflammatory mediators, also modulate cardiac damage (41).

### 3.3. Burn injury causes persistent changes in metabolite profiles reflecting hypermetabolism and hyperglycemia

Acute burn trauma is associated with long-term increased risk of developing multiple morbidities and with greater all-cause mortality (7). Burn injury exerts a profound physiological and metabolic impact resulting in raised resting energy expenditure, inflammation, altered cardiac and organ function and hypermetabolism and these effects persist for several years following the acute injury (6, 42). In the current study, we applied different NMR-based assays and integrated the metabolic response with cytokine data. All three NMR datasets indicated that prior burn injury, sustained 3 years prior to the study, induced a prolonged impact on the plasma phenotype with systematic differences in comparison with the non-injured group. These results are consistent with the known incidence of hypermetabolism and hyperglycemia, which are initiated a few days following injury and continue for several years after the burn event (43, 44), but which are not necessarily associated with the degree or severity of the burn.

We found a trend toward lower mean plasma concentrations of glucose for the control group in comparison with the burn injury group, which contributed to the differential weighting in the CPMG NMR data set (low molecular weight molecules) but was not significant for the univariate comparison using the quantified metabolite data. Burn injury stimulates insulin production and produces insulin resistance in liver, skeletal muscle, and adipose tissue, associated with post-receptor alterations such as phosphorylation of the insulin receptor substrate-1 (IRS-1) in the absence of changes in insulin receptor binding (45). Hyperglycemia during the acute phase of burn injury has been shown to be predictive of clinical outcome with high glucose levels being associated with higher incidence of infection, sepsis, and mortality (46). Similarly, abnormal insulin sensitivity indicative of peripheral and whole-body insulin resistance has been noted.

In this study, we find distinctly higher concentrations of branched chain amino acids (BCAAs) including valine, leucine, isoleucine and aromatic amino acids (AAAs: phenylalanine, tyrosine) in the prior burn injury group. Both branched chain amino acids (BCAAs) and aromatic amino acids (AAAs) have been associated with increased risk of developing type 2 diabetes with various mechanisms being proposed for their role in development of type 2 diabetes (47, 48). Leucine and other amino acids can induce pancreatic beta cells and activate the mammalian target of rapamycin (mTOR) mitogenic signaling pathway, which results in early beta cell dysfunction (49, 50). However, BCAAs can also enhance glycogen production from non-insulin dependent pathways *via* protein kinase C or phosphatidylinositol 3-kinase (51). The upregulation of BCAA and AAA production is consistent with the known 2.21-fold higher increase in developing type 2 diabetes within 5 years post burn injury (52). The subtle elevation of glucose and BCAAs along with the increase in serum phenylalanine levels may be indicative of an underlying impact on insulin sensitivity in the prior burn group.

One of the strongest changes in the burn cohort was the elevation of glutamine and depletion of glutamate, in comparison to healthy controls, with a 2.5-fold higher glutamine:glutamate ratio. In our previous targeted mass spectrometric study, we found similar evidence of hypermetabolism with significantly higher concentrations of 11 amino acids and quinolinic acid. Several of the amino acids overlapped with those measured by ^1^H NMR spectroscopy including alanine, phenylalanine, with several other amino acids demonstrating a similar trend, although not statistically significant (14). In contrast, with our observation of a higher glutamine: glutamate ratio in the prior burn injury group, most of the literature characterizing the metabolic phenotype of metabolic dysregulation [e.g., in relation to obesity (53), type-2 diabetes (54, 55) and recovery from viral infections such as long-COVID (56)] points to higher concentrations of glutamate and a lower glutamine: glutamate ratio driving the metabolic dysfunction. Similarly in incubated enterocytes obtained from burn-injury participants, increased utilization of glutamine, has been shown to lead to the increased formation of glutamate and alanine (57). The difference between the results found in the current study and other literature is difficult to reconcile and is complicated by the fact that most of the literature studies describe adult populations. Therefore the discrepancy in glutamine: glutamate ratio may be influenced by the fact that glutamine and glutamate production differs between adults and children (58).

The generalized hypermetabolic response associated with burn injury is not dependent on the size of burn and the responses elicited by a burn covering 30% or 50% of the total body surface area are the same (48). Jeschke et al. showed that unlike the hyperinflammatory response that persists for around 6 weeks post-injury, the hypermetabolic state and hyperglycemia can persist for several years (5). Here we show that prior burn injury is characterized by higher plasma glucose concentrations for at least 3 years after non-severe burns. Statistical correlation driven from the apex of the α-anomeric glucose signal at δ5.25 showed a weak correlation with GlycA/GlycB peaks (*r* < 0.25) indicating that the hyperglycemia may be associated with associated with inflammation since GlycA has been shown to reflect inflammation and is strongly correlated with high sensitivity C-reactive protein (hsCRP) (49). In fact, GlycA has been suggested to be a more robust marker of inflammation than C-reactive protein (CRP) (19). Following burn injury, it has been shown that metabolism shifts toward increased glycolysis, glycogenolysis, gluconeogenesis, lipolysis and proteolysis resulting from a severe energy deprivation at the cellular level promoting a hypermetabolic state (50). Altered hormone levels (increased circulating cortisol, catecholamines and glucagon) following burn injury also contribute to the hypermetabolic state (4) and serve to increase the cycling of glucose and free fatty acids (36).

Hypermetabolism induced by thermal injury has been associated with a switch from white to beige or brown fat metabolism, which may be related to increased mitochondrial mass and uncoupling protein 1 expression (59). Consistent with disrupted mitochondrial metabolism, we find prior burn injury to be associated upregulation of the tricarboxylic acid cycle as indicated by significantly higher concentrations of citrate in the multivariate models. Elevated pyruvic acid concentrations in the prior burn injury group may also impact on altered mitochondrial metabolism and has previously been reported to be increased 7 days post burn (60). It has been suggested that following burn injury, pyruvate is diverted from the TCA cycle toward lactate and alanine synthesis with Valbuena et al. finding that 60% of pyruvate, lactate and alanine carbons was glucose-derived following burn injury (61). Here we see increased plasma pyruvate and alanine without a concomitant increase in lactate concentrations. Another theory, proposed by Wolfe et al., is that there is an increased rate of substrate recycling in burn patients resulting in enhanced glycolytic-gluconeogenic and triglyceride-fatty acid cycling, which could serve to provide more metabolic flexibility to adapt efficiently to changes in burn-induced energy-substrate demands (62). Part of this energy recycling involves an upregulation of the Cahill cycle, which serves the purpose of transporting toxic nitrogenous waste from the muscles to the liver by converting L-glutamate and pyruvate into α-ketoglutarate and L-alanine (63), facilitating the transport of alanine to the liver where alanine is oxidatively deaminated to form pyruvate. The levels of alanine and aspartate aminotransferases have been shown to increase post-acute burn injury. However, Chiarelli et al. found that these transaminase levels do not always drop immediately after acute injury (64).

Phenylalanine concentrations were significantly higher in the plasma of the burn injury group than controls. Higher levels of phenylalanine have been noted in association with burn injury (primarily in the acute phase) and the elevation was associated with the hypermetabolic state (65). In severe cases of burn, even though the total free amino acids in plasma dropped, plasma phenylalanine and the phenylalanine to tyrosine ratio was consistently higher and was strongly associated with death and weight loss in both animals and patients (66). With regard to the current study, in the burn group, but not the control, phenylalanine correlated positively with multiple cytokines and small low density lipoprotein particles but negatively with 3-D-hydroxybutyrate. Collectively, there was a reduction of plasma ketone bodies in the burn injury group, although this was only significant for 3-D-hydroxybutyrate (Supplementary Figure S3A). Abbott et al. observed that in the initial phase of recovery from burn injury, patients failed to mount a ketonemic response to starvation monitored in non-burn participants following starvation (67).

### 3.4. Study strengths and limitations

This study utilizes novel metabolic profiling strategies to interrogate cardiometabolic risk in an overlooked vulnerable population. Here, we have demonstrated the elevated cardiometabolic risk for young children who have previously suffered from a burn injury. While prior research has established that these individuals are disproportionately affected by the development of comorbidities later in life, few follow up studies such as this exist. Although this current study was statistically underpowered for assessing if there are metabolic differences between different sources of burn, we did observe a difference between scald and flame burns scalds versus flame in the LDL lipoprotein signature with the L6PL subfraction associating with flame burns and lower density triglyceride subfractions associating with scalds. In a mouse model of thermal injury, IFN-γ was reported to be higher in scald-burned mice than in sham- and flame-burned mice suggesting that the type of burn injury may exert a differential effect on the immune-metabolic phenotypes (68). Given that there is a different chemical background to flame versus scald burns, with flame burns inducing greater formation of new protein degradation products, it is feasible that the systemic metabolic response may differ and warrants further investigation in a larger cohort. The authors recognize the limitations and constraints of capturing metabolic profiles at a single timepoint, and thus follow-up studies will be necessary.

## Conclusion

The integrated multi-modal signature of plasma samples was indicative of chronic hypermetabolic and hyperinflammatory processes persisting at least 3 years post burn injury, driven by an immuno-metabolic memory. These processes were evident in the metabolic, glycoprotein, lipoprotein, and cytokine profiles. The weighted linkages between the various molecular panels underscored the systemic response to inflammation suggesting that the body mounts a prolonged response to the initial trauma with co-ordinated response between the small dense lipoprotein particles and a Th2-dominated cytokine profile. These findings support prior observation of a chronic hypermetabolic state following acute injury, driven by an immuno-metabolic memory. Further investigation of this systems response may uncover new understanding of the association of burn injury with increased long-term morbidity and mortality.

## Data availability statement

The datasets presented in this study can be found in online repositories. The names of the repository/repositories and accession number(s) can be found at: https://zenodo.org/, accession number: 7344673.

## Ethics statement

The studies involving human participants were reviewed and approved by Child and Adolescent Health Service WA (approval numbers: 2015219EP; 1111EP; 768EP). Written informed consent to participate in this study was provided by the participants’ legal guardian/next of kin.

## Author contributions

SB: conceptualization, data curation, formal analysis, data visualization and manuscript preparation. SL and BJ: investigation, methodology, and manuscript preparation. DH, LW, and NG: investigation and methodology. SHB: investigation, methodology, and resources. VF: funding acquisition and manuscript preparation. MF and FW: conceptualization and manuscript preparation. EH and JN: conceptualization, funding acquisition, formal analysis, and manuscript preparation. All authors contributed to the article and approved the submitted version.

## Funding

MF is supported by the Stan Perron Center of Excellence in Childhood Burns and the Perth Children’s Hospital Foundation. MRFF for funding the Australian National Phenome Center for development of methods used in this research. We thank the UK Medical Research Council (MRC), the Imperial College London MRC Doctoral Training Program and the Imperial College London Dean’s Award for funding SB, the Department of Jobs, Tourism, Science and Innovation, Government of Western Australian Premier’s Fellowship for funding EH, and the ARC Laureate Fellowship funding for EH.

## Conflict of interest

The authors declare that the research was conducted in the absence of any commercial or financial relationships that could be construed as a potential conflict of interest.

## Publisher’s note

All claims expressed in this article are solely those of the authors and do not necessarily represent those of their affiliated organizations, or those of the publisher, the editors and the reviewers. Any product that may be evaluated in this article, or claim that may be made by its manufacturer, is not guaranteed or endorsed by the publisher.

## Abbreviations

TBSA: Total Body Surface Area
NMR: Nuclear Magnetic Resonance
CPMG: Carr-Purcell Meiboom-Gill
JEDI-PGPE: J-Edited DiFFusional Pulsed Gradient Echo Experiment
PCA: Principal Component Analysis
OPLS-DA: Orthogonal Partial Least Squares-Discriminant Analysis
SD: Standard Deviation
GlycA: α-1-acid glycoprotein signal A
GlycB: α-1-acid glycoprotein signal B
SPC: Supramolecular Phospholipid Composite
STOCSY: Statistical TOtal Correlation SpectroscopY
