# Supplementary material for: Cardiometabolic disease risk markers are increased following burn injury in children
Source: Front Public Health. 2023 Jun 2;11:1105163. doi: 10.3389/fpubh.2023.1105163 (PMC10275366; doi:10.3389/fpubh.2023.1105163)
Supplement: Supplementary file 1 [file Table_1.DOCX]

**Supplementary Materials and Methods**

**Supplementary Table 1: Abbreviations and annotation of Lipoproteins from the Bruker iVDR B.I.-LISA^TM^ method.**

**Abbreviations:** LDL – low-density lipoprotein; HDL – high-density lipoprotein; VLDL – very low-density lipoprotein; IDL – intermediate-density lipoprotein.

| **Key** | **Class/subclass** | **Compound** | **Concentration unit** |
| --- | --- | --- | --- |
| **TPTG** | Total Plasma | Triglycerides | mg/dL |
| **TPCH** | Total Plasma | Cholesterol | mg/dL |
| **LDCH** | LDL | Cholesterol | mg/dL |
| **HDCH** | HDL | Cholesterol | mg/dL |
| **TPA1** | Total Plasma | Apolipoprotein-A1 | mg/dL |
| **TPA2** | Total Plasma | Apolipoprotein-A2 | mg/dL |
| **TPAB** | Total Plasma | Apolipoprotein-B100 | mg/dL |
| **LDHD** | Ratio LDL and HDL | LDL Cholesterol / HDL | -/- |
|  | Cholesterol | Cholesterol |  |
| **ABA1** | Ratio of | Apolipoprotein-A1 / Apolipoprotein-B100 | -/- |
|  | Apolipoproteins A1 and B100 |  |  |
| **TBPN** | Apolipoprotein-B100 | Particle Number | nmol/L |
|  | carrying particles |  |  |
| **VLPN** | VLDL | Particle Number | nmol/L |
| **IDPN** | IDL | Particle Number | nmol/L |
| **LDPN** | LDL | Particle Number | nmol/L |
| **L1PN** | LDL-1 | Particle Number | nmol/L |
| **L2PN** | LDL-2 | Particle Number | nmol/L |
| **L3PN** | LDL-3 | Particle Number | nmol/L |
| **L4PN** | LDL-4 | Particle Number | nmol/L |
| **L5PN** | LDL-5 | Particle Number | nmol/L |
| **L6PN** | LDL-6 | Particle Number | nmol/L |
| **VLTG** | VLDL Class | Triglycerides | mg/dL |
| **IDTG** | IDL Class | Triglycerides | mg/dL |
| **LDTG** | LDL Class | Triglycerides | mg/dL |
| **HDTG** | HDL Class | Triglycerides | mg/dL |
| **VLCH** | VLDL Class | Cholesterol | mg/dL |
| **IDCH** | IDL Class | Cholesterol | mg/dL |
| **LDCH** | LDL Class | Cholesterol | mg/dL |
| **HDCH** | HDL Class | Cholesterol | mg/dL |
| **VLFC** | VLDL Class | Free Cholesterol | mg/dL |
| **IDFC** | IDL Class | Free Cholesterol | mg/dL |
| **LDFC** | LDL Class | Free Cholesterol | mg/dL |
| **HDFC** | HDL Class | Free Cholesterol | mg/dL |
| **VLPL** | VLDL Class | Phospholipids | mg/dL |
| **IDPL** | IDL Class | Phospholipids | mg/dL |
| **LDPL** | LDL Class | Phospholipids | mg/dL |
| **HDPL** | HDL Class | Phospholipids | mg/dL |
| **HDA1** | HDL Class | Apolipoprotein-A1 | mg/dL |
| **HDA2** | HDL Class | Apolipoprotein-A2 | mg/dL |
| **VLAB** | VLDL Class | Apolipoprotein-B100 | mg/dL |
| **IDAB** | IDL Class | Apolipoprotein-B100 | mg/dL |
| **LDAB** | LDL Class | Apolipoprotein-B100 | mg/dL |
| **V1TG** | VLDL-1 Subclass | Triglycerides | mg/dL |
| **V2TG** | VLDL-2 Subclass | Triglycerides | mg/dL |
| **V3TG** | VLDL-3 Subclass | Triglycerides | mg/dL |
| **V4TG** | VLDL-4 Subclass | Triglycerides | mg/dL |
| **V5TG** | VLDL-5 Subclass | Triglycerides | mg/dL |
| **V1CH** | VLDL-1 Subclass | Cholesterol | mg/dL |
| **V2CH** | VLDL-2 Subclass | Cholesterol | mg/dL |
| **V3CH** | VLDL-3 Subclass | Cholesterol | mg/dL |
| **V4CH** | VLDL-4 Subclass | Cholesterol | mg/dL |
| **V5CH** | VLDL-5 Subclass | Cholesterol | mg/dL |
| **V1FC** | VLDL-1 Subclass | Free Cholesterol | mg/dL |
| **V2FC** | VLDL-2 Subclass | Free Cholesterol | mg/dL |
| **V3FC** | VLDL-3 Subclass | Free Cholesterol | mg/dL |
| **V4FC** | VLDL-4 Subclass | Free Cholesterol | mg/dL |
| **V5FC** | VLDL-5 Subclass | Free Cholesterol | mg/dL |
| **V1PL** | VLDL-1 Subclass | Phospholipids | mg/dL |
| **V2PL** | VLDL-2 Subclass | Phospholipids | mg/dL |
| **V3PL** | VLDL-3 Subclass | Phospholipids | mg/dL |
| **V4PL** | VLDL-4 Subclass | Phospholipids | mg/dL |
| **V5PL** | VLDL-5 Subclass | Phospholipids | mg/dL |
| **L1TG** | LDL-1 Subclass | Triglycerides | mg/dL |
| **L2TG** | LDL-2 Subclass | Triglycerides | mg/dL |
| **L3TG** | LDL-3 Subclass | Triglycerides | mg/dL |
| **L4TG** | LDL-4 Subclass | Triglycerides | mg/dL |
| **L5TG** | LDL-5 Subclass | Triglycerides | mg/dL |
| **L6TG** | LDL-6 Subclass | Triglycerides | mg/dL |
| **L1CH** | LDL-1 Subclass | Cholesterol | mg/dL |
| **L2CH** | LDL-2 Subclass | Cholesterol | mg/dL |
| **L3CH** | LDL-3 Subclass | Cholesterol | mg/dL |
| **L4CH** | LDL-4 Subclass | Cholesterol | mg/dL |
| **L5CH** | LDL-5 Subclass | Cholesterol | mg/dL |
| **L6CH** | LDL-6 Subclass | Cholesterol | mg/dL |
| **L1FC** | LDL-1 Subclass | Free Cholesterol | mg/dL |
| **L2FC** | LDL-2 Subclass | Free Cholesterol | mg/dL |
| **L3FC** | LDL-3 Subclass | Free Cholesterol | mg/dL |
| **L4FC** | LDL-4 Subclass | Free Cholesterol | mg/dL |
| **L5FC** | LDL-5 Subclass | Free Cholesterol | mg/dL |
| **L6FC** | LDL-6 Subclass | Free Cholesterol | mg/dL |
| **L1PL** | LDL-1 Subclass | Phospholipids | mg/dL |
| **L2PL** | LDL-2 Subclass | Phospholipids | mg/dL |
| **L3PL** | LDL-3 Subclass | Phospholipids | mg/dL |
| **L4PL** | LDL-4 Subclass | Phospholipids | mg/dL |
| **L5PL** | LDL-5 Subclass | Phospholipids | mg/dL |
| **L6PL** | LDL-6 Subclass | Phospholipids | mg/dL |
| **L1AB** | LDL-1 Subclass | Apolipoprotein-B100 | mg/dL |
| **L2AB** | LDL-2 Subclass | Apolipoprotein-B100 | mg/dL |
| **L3AB** | LDL-3 Subclass | Apolipoprotein-B100 | mg/dL |
| **L4AB** | LDL-4 Subclass | Apolipoprotein-B100 | mg/dL |
| **L5AB** | LDL-5 Subclass | Apolipoprotein-B100 | mg/dL |
| **L6AB** | LDL-6 Subclass | Apolipoprotein-B100 | mg/dL |
| **H1TG** | HDL-1 Subclass | Triglycerides | mg/dL |
| **H2TG** | HDL-2 Subclass | Triglycerides | mg/dL |
| **H3TG** | HDL-3 Subclass | Triglycerides | mg/dL |
| **H4TG** | HDL-4 Subclass | Triglycerides | mg/dL |
| **H1CH** | HDL-1 Subclass | Cholesterol | mg/dL |
| **H2CH** | HDL-2 Subclass | Cholesterol | mg/dL |
| **H3CH** | HDL-3 Subclass | Cholesterol | mg/dL |
| **H4CH** | HDL-4 Subclass | Cholesterol | mg/dL |
| **H1FC** | HDL-1 Subclass | Free Cholesterol | mg/dL |
| **H2FC** | HDL-2 Subclass | Free Cholesterol | mg/dL |
| **H3FC** | HDL-3 Subclass | Free Cholesterol | mg/dL |
| **H4FC** | HDL-4 Subclass | Free Cholesterol | mg/dL |
| **H1PL** | HDL-1 Subclass | Phospholipids | mg/dL |
| **H2PL** | HDL-2 Subclass | Phospholipids | mg/dL |
| **H3PL** | HDL-3 Subclass | Phospholipids | mg/dL |
| **H4PL** | HDL-4 Subclass | Phospholipids | mg/dL |
| **H1A1** | HDL-1 Subclass | Apolipoprotein-A1 | mg/dL |
| **H2A1** | HDL-2 Subclass | Apolipoprotein-A1 | mg/dL |
| **H3A1** | HDL-3 Subclass | Apolipoprotein-A1 | mg/dL |
| **H4A1** | HDL-4 Subclass | Apolipoprotein-A1 | mg/dL |
| **H1A2** | HDL-1 Subclass | Apolipoprotein-A2 | mg/dL |
| **H2A2** | HDL-2 Subclass | Apolipoprotein-A2 | mg/dL |
| **H3A2** | HDL-3 Subclass | Apolipoprotein-A2 | mg/dL |
| **H4A2** | HDL-4 Subclass | Apolipoprotein-A2 | mg/dL |

**
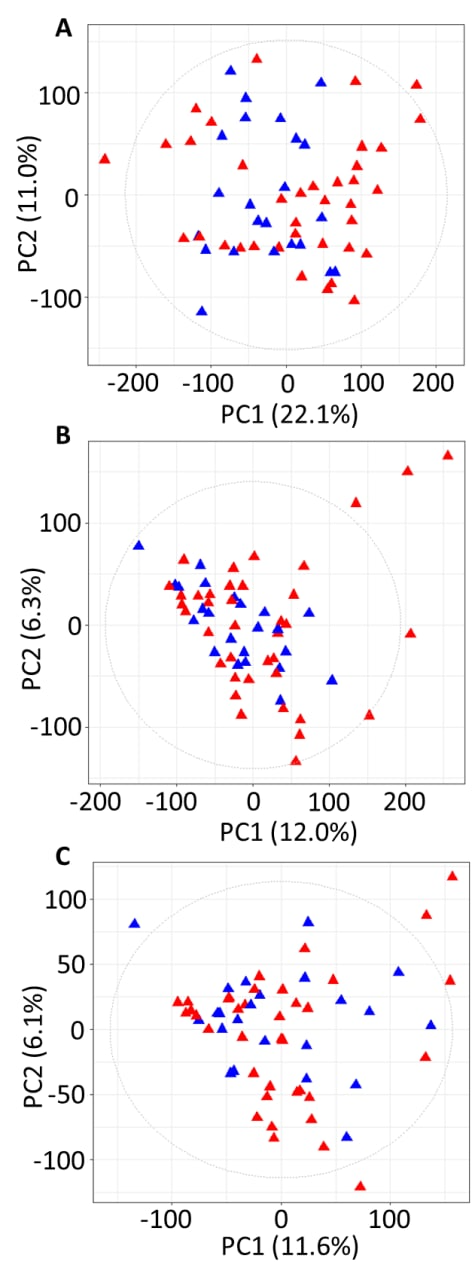
**

**Supplementary Figure S1:** Scores plot of PCA models from A) standard 1D, B) CPMG, C) JEDI-PGPE NMR spectra.


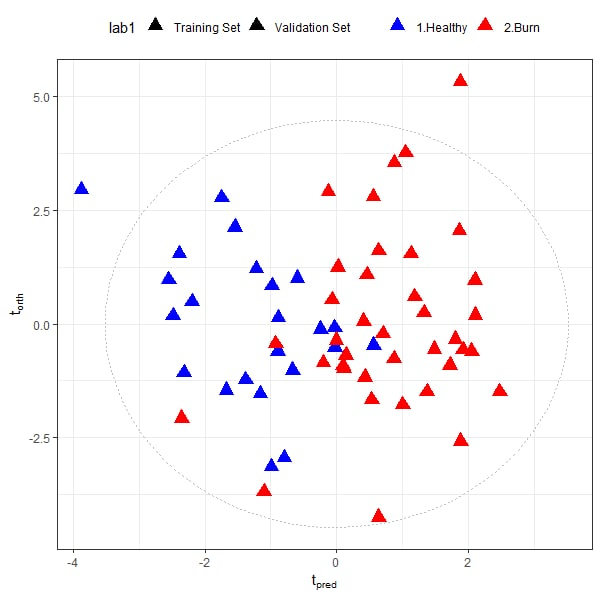
**Supplementary Figure S2A:** Scores plot of the OPLS-DA model constructed using B.I.-LISA^TM^ quantified small molecules (R^2^X 0.12, AUROC 0.95, CV-AUROC 0.81).


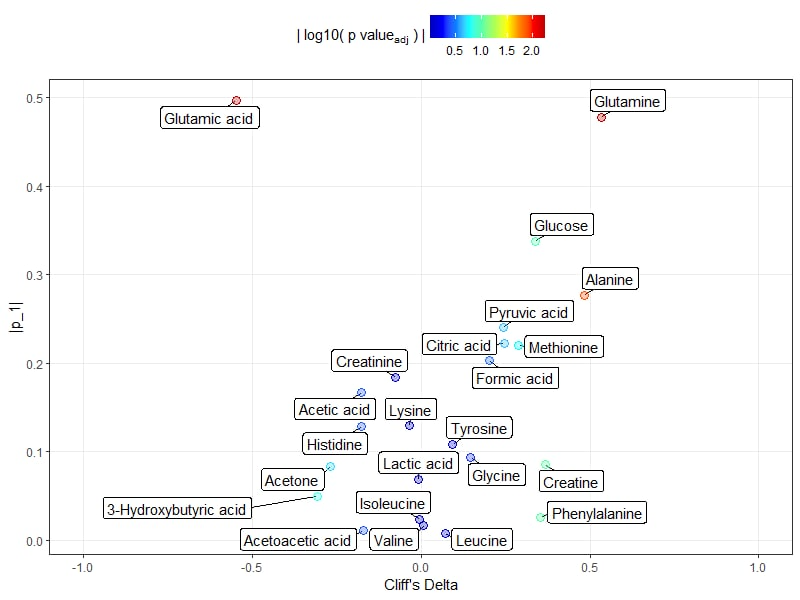


**Supplementary Figure S2B:** Variable importance eruption plot of predictive small molecule concentrations based on the OPLS-DA component loadings (P_pred_) and univariate effect sizes (Cliff’s delta). Co-ordinates are coloured by the FDR-adjusted *p-*value (<0.05) from the statistical group comparison.


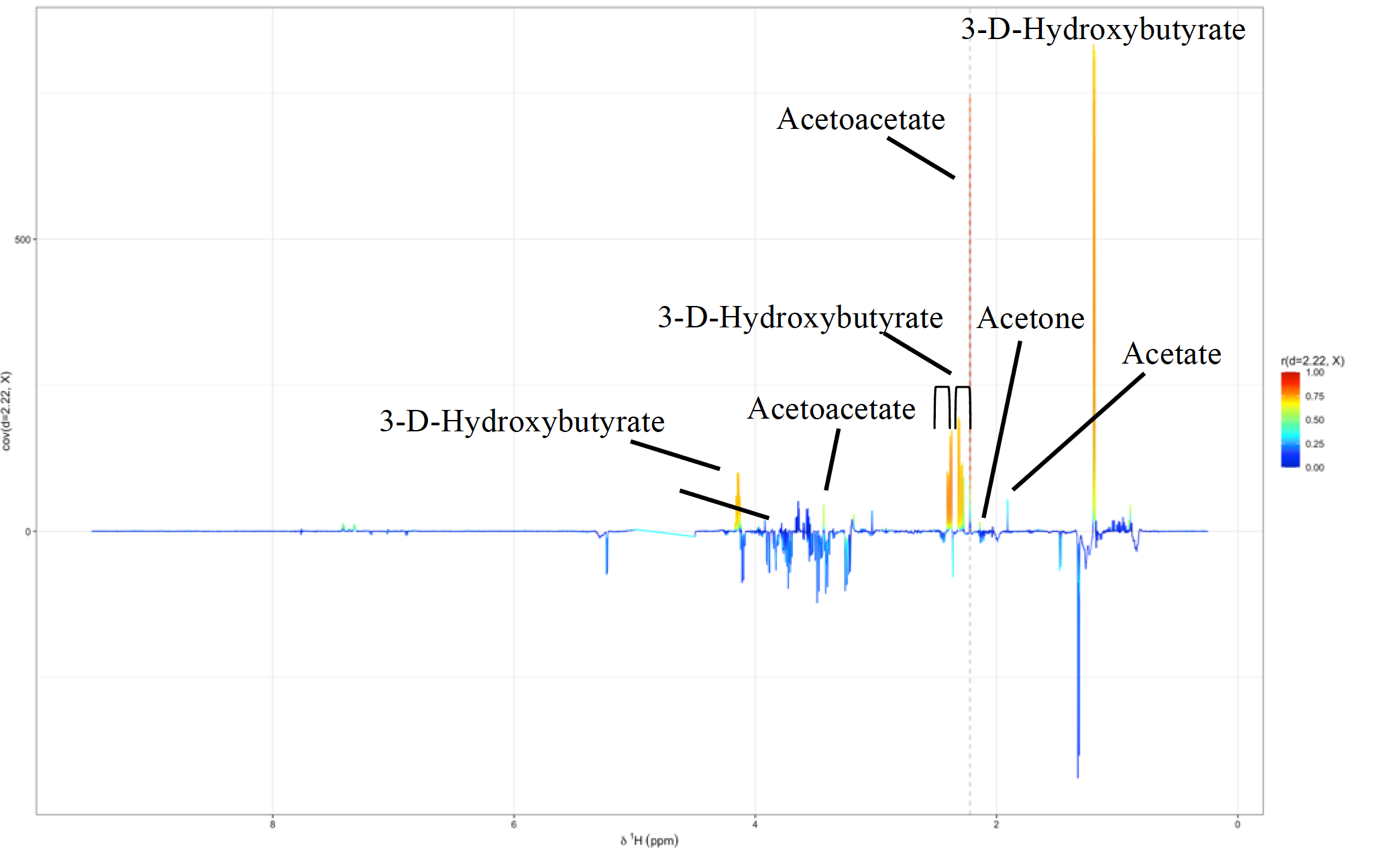


**Supplementary Figure S3A:** Statisical Total COrrelation SpectroscopY (STOCSY) plot presenting correlation profiles for acetoacetate (ð2.22).


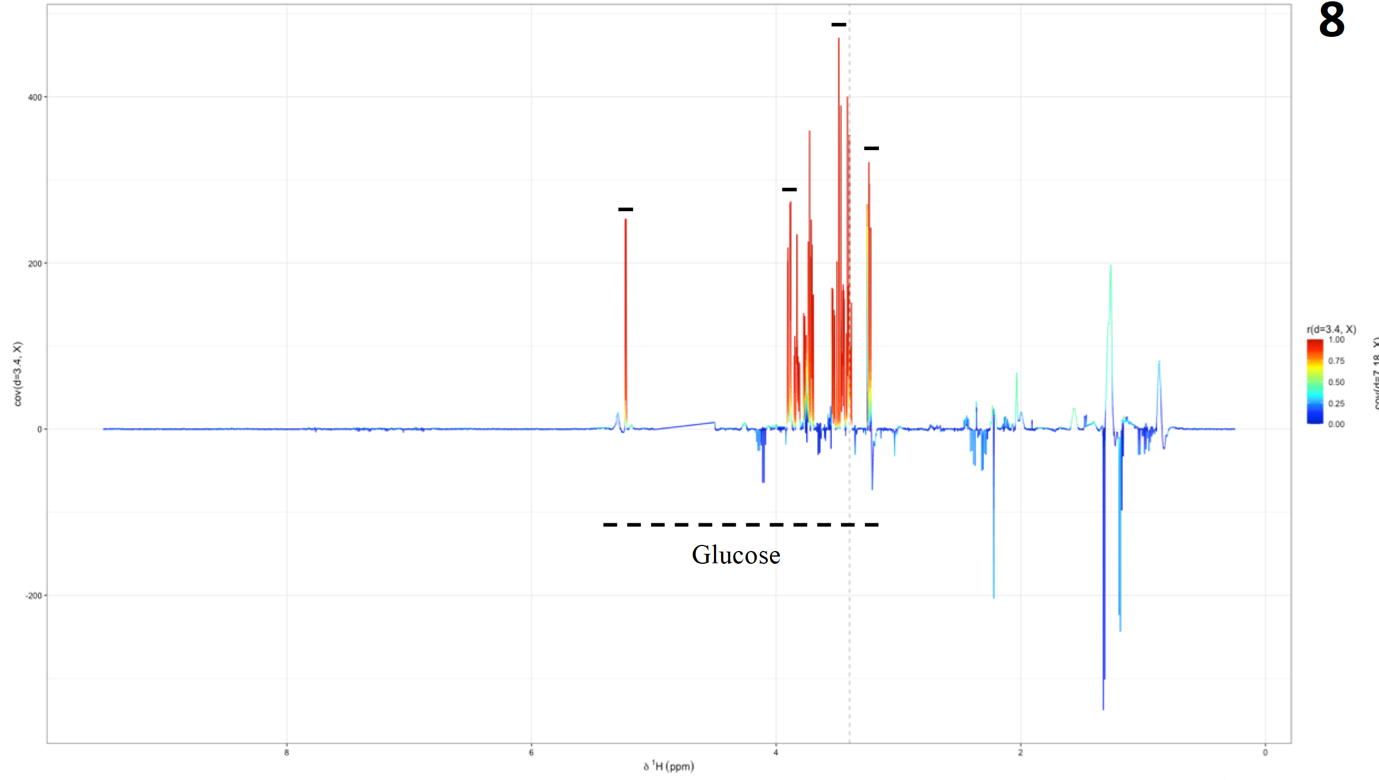


**Supplementary Figure S3B:** Statisical Total COrrelation SpectroscopY (STOCSY) plot presenting correlation profiles for glucose (ð3.4).


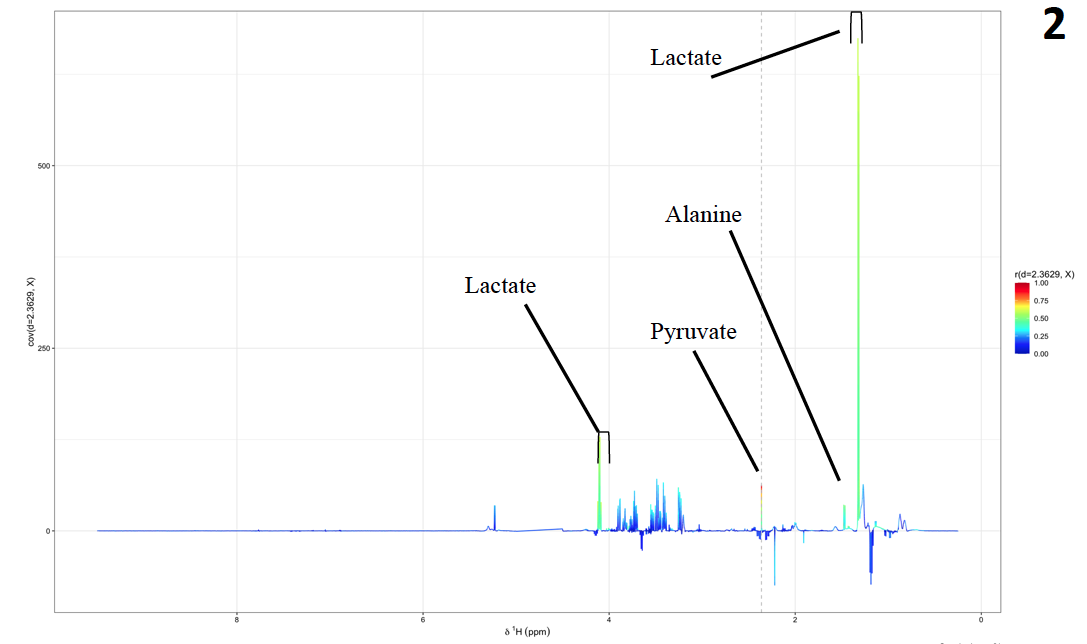


**Supplementary Figure S3C:** Statisical Total COrrelation SpectroscopY (STOCSY) plot presenting correlation profiles for pyruvate (ð2.3629).
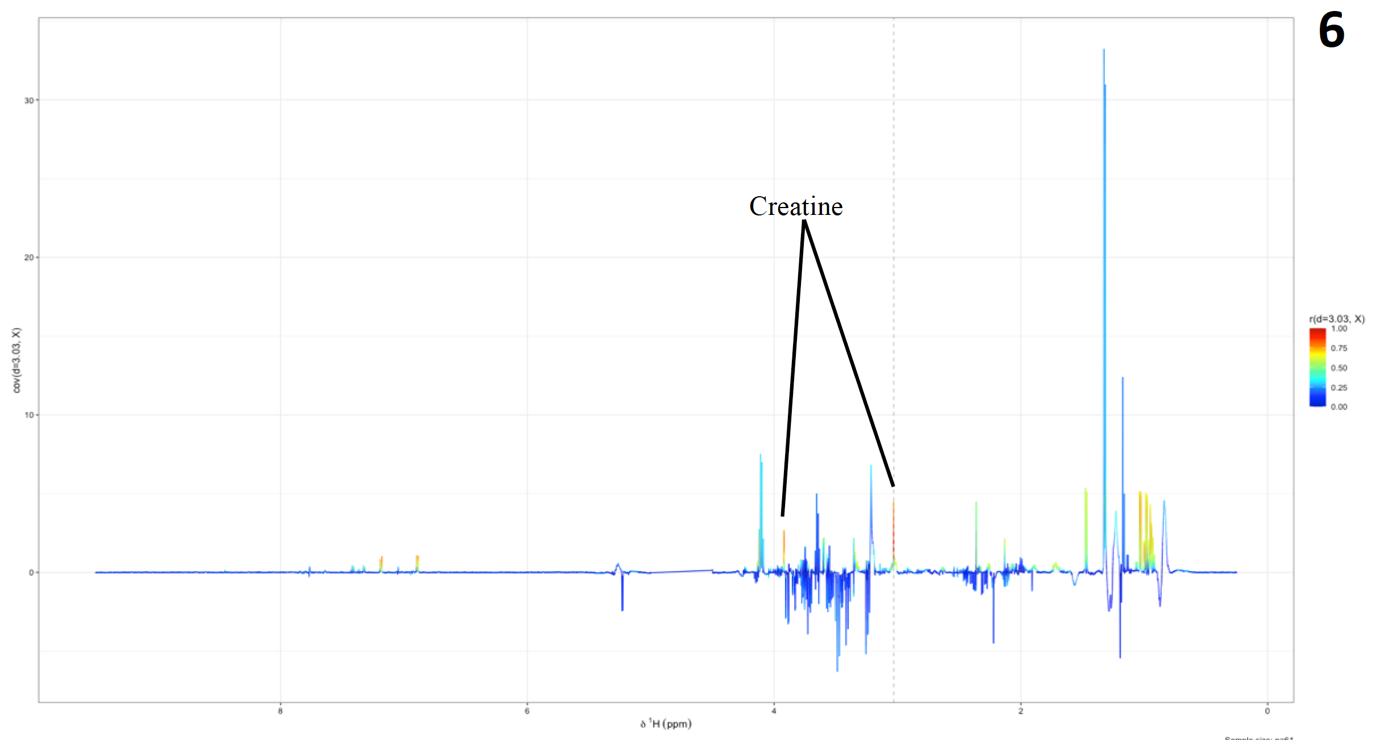


**Supplementary Figure S3D:** Statisical Total COrrelation SpectroscopY (STOCSY) plot presenting correlation profiles for creatine (ð3.03).


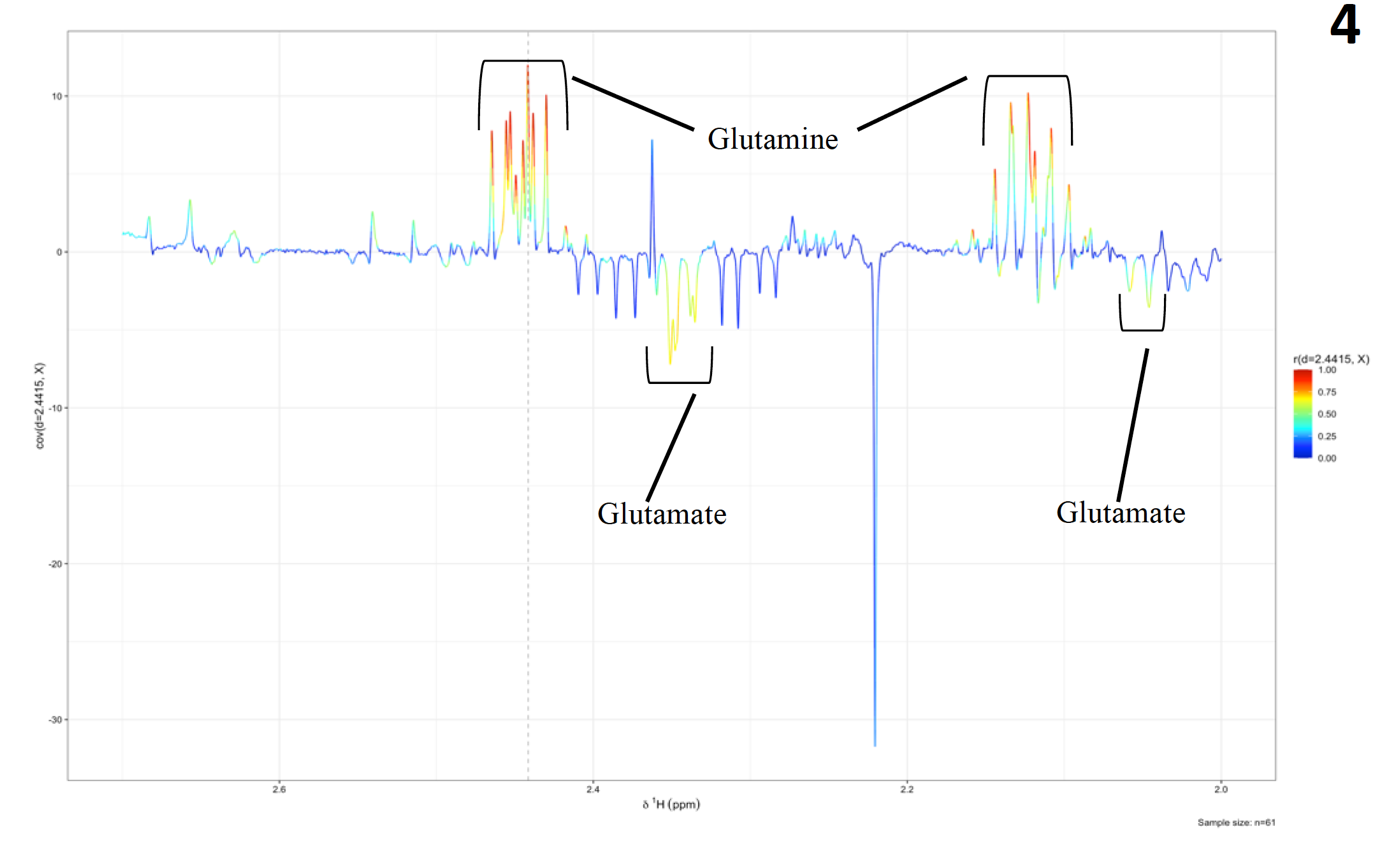


**Supplementary Figure S3E:** Statisical Total COrrelation SpectroscopY (STOCSY) plot presenting correlation profiles for glutamine (ð2.4415).


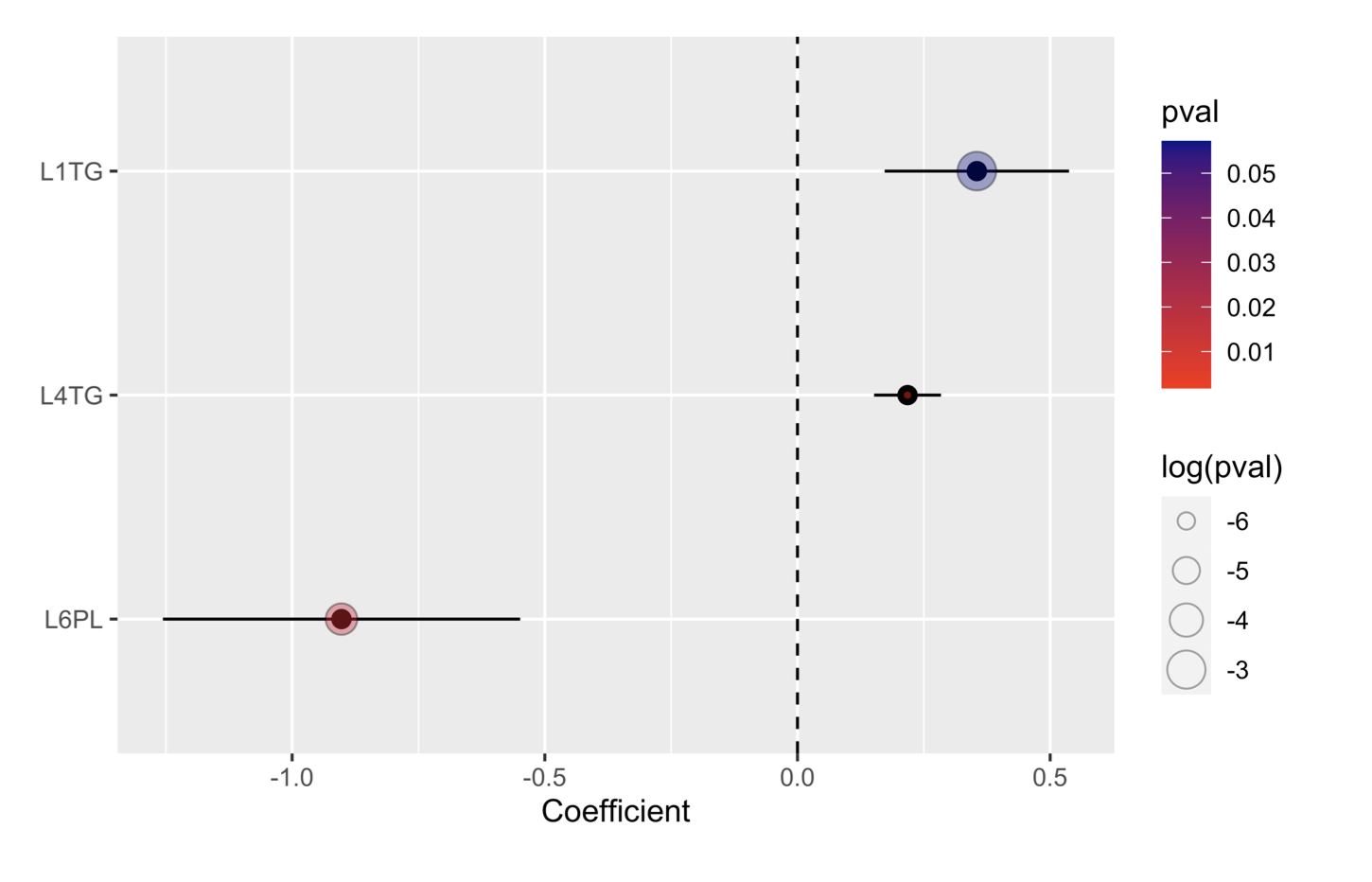
**Supplementary Figure S4**: Multivariate linear regression coefficients of significantly different lipoprotein parameters for source of burn injury phenotype: scald (positive beta coefficient) versus flame (negative beta coefficient).

**B**

**A**


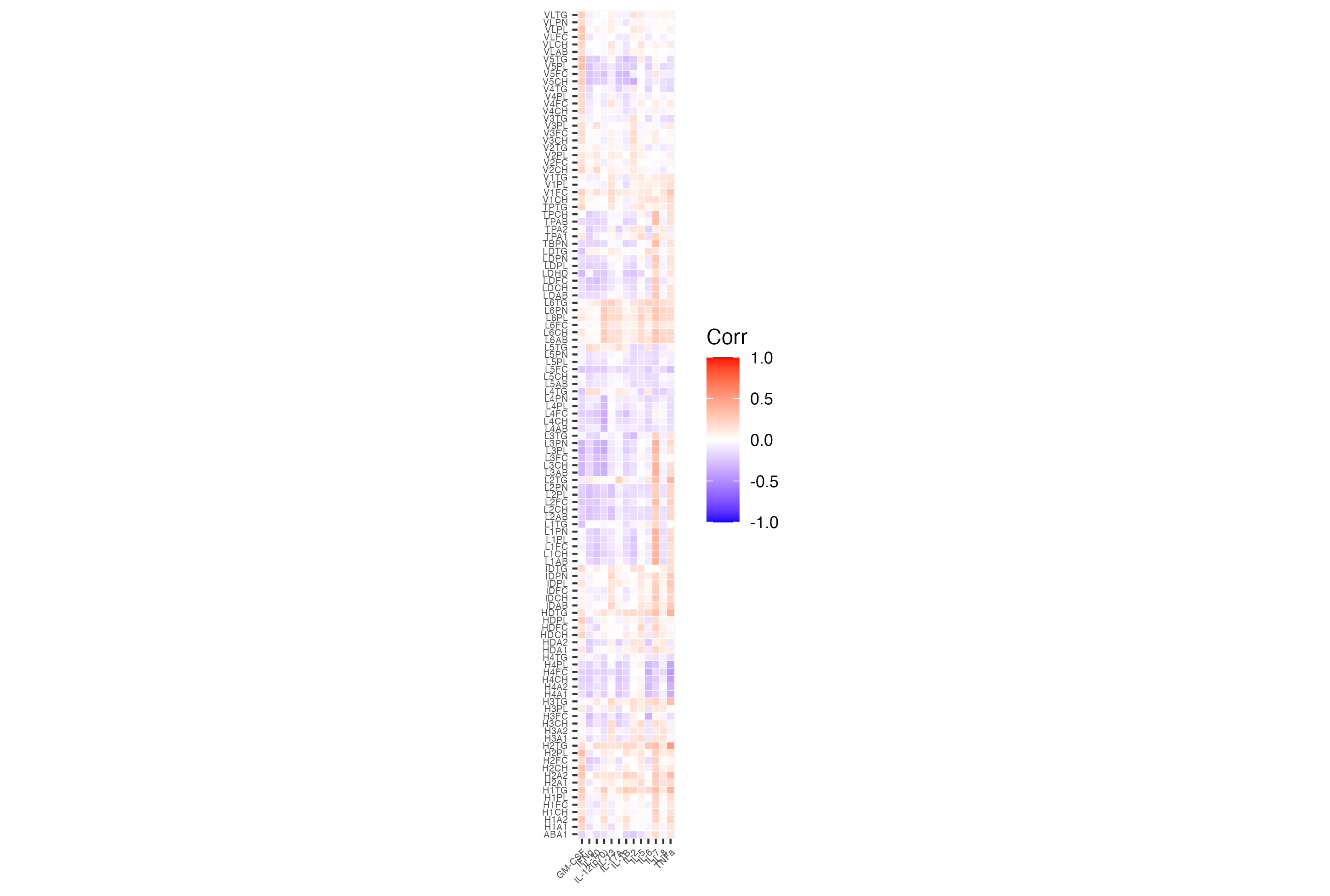


Burn Injury


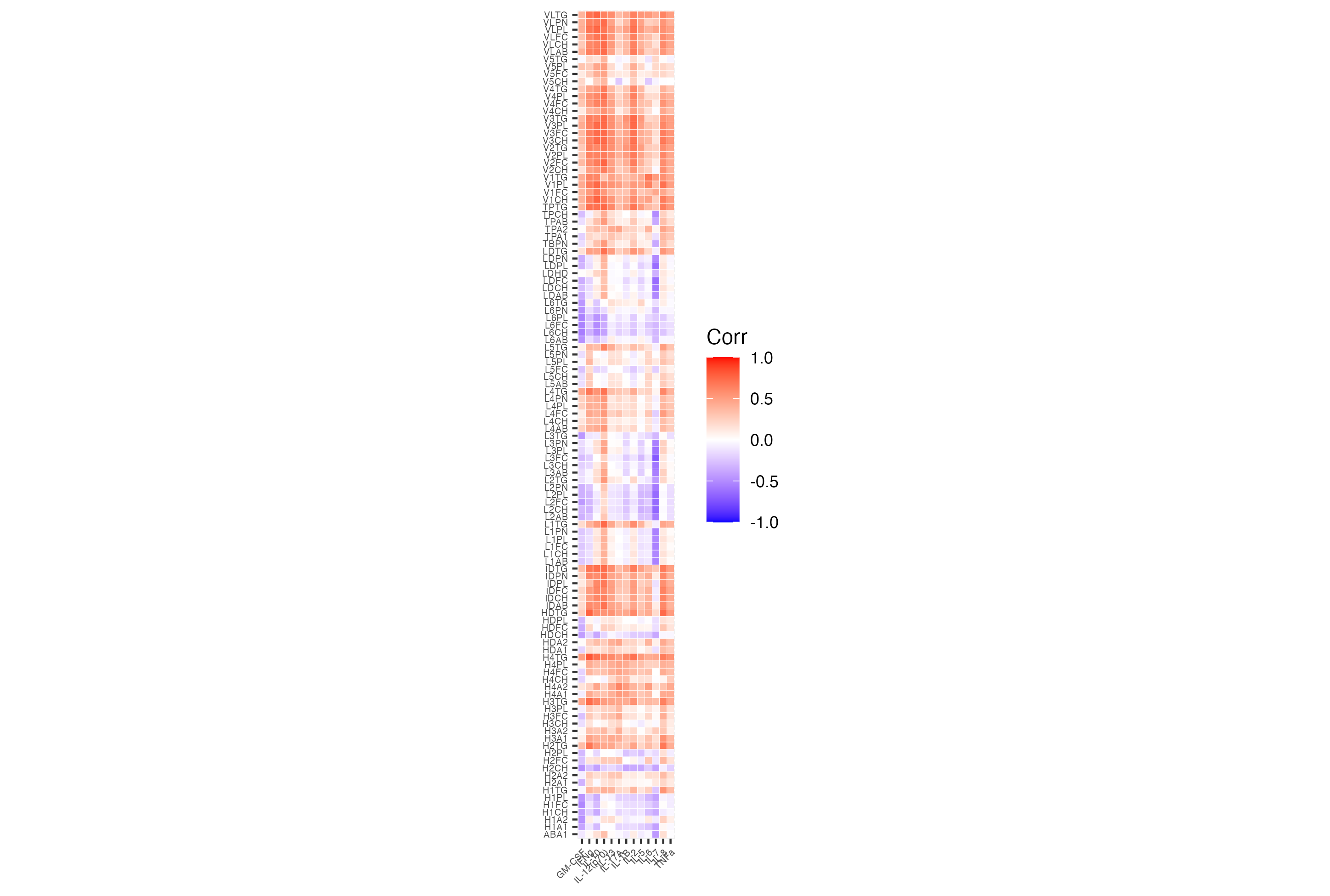


Non-Burn Control

**Supplementary Figure S5:** Lipoprotein - cytokine spearman’s correlations. Quantified lipoproteins and cytokines correlated against one another, showing the signature present in A) non-burn controls and B) burn injury children.

**Supplementary Figure S6:** PCA scores plot constructed from combined quantified small molecule and lipoprotein data sets demonstrating the different phenotypes within the burn injury group.
