# Supplementary material for: Cardiometabolic disease risk markers are increased following burn injury in children
Source: Front Public Health. 2023 Jun 2;11:1105163. doi: 10.3389/fpubh.2023.1105163 (PMC10275366; doi:10.3389/fpubh.2023.1105163)
Supplement: Supplementary file 2 [file Table_2.docx]

**STAR★Methods**

**Resource Availability**

**Key resources table**

| **REAGENT or RESOURCE** | **SOURCE** | **IDENTIFIER** |
| --- | --- | --- |
| **Biological samples** | | |
| Plasma | WA Paediatric Burns Biobank | N/A (MTA) |
| Long term reference plasma | PM separation | SP2000 |
| **Chemicals, peptides, and recombinant proteins** | | |
| 75 mM Na2HPO4 | Bruker Biospin | N/A |
| 2 mM NaN3 | Bruker Biospin | N/A |
| 4.6 mM sodium trimethylsilyl propionate-[2,2,3,3-2H4] (TSP) | Bruker Biospin | N/A |
| D2O | Cambridge Isotope Laboratories | N/A |
| **Software and algorithms** | | |
| Bruker TOPSPIN 3 | Bruker Biospin | N/A |

**Lead Contact**

Further information and requests for resources and reagents should be directed to and will be fulfilled by the lead contact, Jeremy Nicholson (jeremy.nicholson@imperial.ac.uk).

**Materials availability**

This study did not generate new unique reagents.

**Experimental model and subject details**

**Paediatric Burns Study**

The Paediatric Burns Biobank pilot study recruited 36 children at least 3 years post non severe burn injury, aged 0-4 years of age at the original time of injury and trauma presentation and between 4-8 years at the time of recruitment. Cause of burn was varied across the 36 children: scald (n=23), friction (n=9) and flame (n=4). All recruited patients had no history of pre-existing illness and no medication at time of biobanking. A cohort of 21 non-injured age and sex matched healthy controls were recruited from the general population, with no prior medical history of note. No individual from either group had recent history of acute infection or was on medication at the time of enrolment. Informed consent was obtained for all participants and ethical approval was obtained from the Child and Adolescent Health Service WA (approval numbers: 2015219EP; 1111EP; 768EP). For each participant, blood was collected into tubes containing preservative-free heparin, then centrifuged to collect the plasma, and immediately frozen at -80°C. This study utilises pre-collected biobank samples.

**Cytokine measurements**

Circulating cytokine and levels were analysed and quantified for 30 non-severe burn injury children and 16 age and sex matched non-burn healthy controls, using multiplex immunoassays and cell profiles measured against a panel of 40 metal-conjugated antibodies and mass cytometry and have been reported previously^1^. Briefly, a custom Milliplex MAP human high sensitivity T cell panel multiplex bead assay (MERCK) was used to quantify a panel of cytokines and chemokines: Tumour necrosis factor- alpha (TNF-α), Interleukin-8 (IL-8), Interleukin 7 (IL-7), Interleukin-6 (IL-6), Interleukin-5 (IL-5), Interleukin-2 (IL-2), Interleukin-1beta (IL-1β), Interleukin-17A (IL-17A), Interleukin-13 (IL-13), Interleukin-12 p70 (IL-12(p70)), Interleukin-10 (IL-10), Interferon-gamma (IFN-γ) and Granulocyte macrophage colony stimulating factor (GM-CSF).

**^1^H NMR spectroscopy of plasma samples**

^1^H NMR spectroscopy of plasma was completed according to a previously published method^2^, briefly described as follows: The plasma supernatant was mixed with 75 mM pH 7.4 sodium phosphate, buffer in 1:1 ratio, and 600 µL were transferred into a Bruker SampleJetTM 5mm NMR tube. For quality control, long term plasma reference samples were transferred into a 5 mm SampleJetTM NMR tube using the prior method. NMR analysis was performed on a 600 MHz Bruker Avance III HD spectrometer, with a BBI probe and fitted with an automated Bruker SampleJetTM robot cooling system set to 5°C. Experiments were completed according to the Bruker in vitro Diagnostics research (IVDr) methods. For each sample, three experiments were run in tandem with a total analysis time of 12.5 minutes; ^1^ H 1D experiment with solvent pre-saturation^3^ (32 scans, 98304 data points, spectral width of 18028.85 Hz), a 1D Carr-Purcell-Meiboom-Gill (CPMG) spin-echo experiment (32 scans, 73728 data points, spectral width of 12019.23Hz) and lastly a J-Edited DiFFusional Pulsed Gradient Echo Experiment (JEDI-PGPE) experiment ^4^ (64 scans, 98304 data points, spectral width of 18028.85 Hz). The 1D ^1^H and CPMG spectra were processed in automation using Bruker TopspinTM 3.6.2, and ICON^TM^ NMR for phasing, baseline correction and TSP calibration (δ=0). The PGPE spectra were processed in automation using Bruker TopspinTM 3.6.2, and ICON^TM^ NMR for phasing and baseline correction.

**Lipoprotein subfraction analysis**

A total of 112 lipoprotein parameters were quantified for each sample, using the Bruker IVDr Lipoprotein Subclass Analysis (B.I.-LISA^TM^) method^5^. This is completed by mathematically interrogating and quantifying the -CH_2_ (1.25 ppm) and -CH_3_ (0.8 ppm) peaks of the 1D spectrum after normalization to the Bruker QuantRef TM manager within Topspin^TM^ using a PLS-2 regression model. The various lipoprotein subclasses included different fractions of intermediate-density lipoprotein (IDL, density 1.006–1.019 kg/L), very low-density lipoprotein (VLDL, 0.950–1.006 kg/L), low-density lipoprotein (LDL, density 1.09–1.63 kg/L), and high-density lipoprotein (HDL, density 1.063–1.210 kg/L). The LDL sub-fraction was stratified into six density classes (LDL-1 1.019–1.031 kg/L, LDL-2 1.031–1.034 kg/L, LDL-3 1.034–1.037 kg/L, LDL-4 1.037–1.040 kg/L, LDL-5 1.040–1.044 kg/L, LDL-6 1.044–1.063 kg/L), and the HDL sub-fractions into four density classes (HDL-1 1.063–1.100 kg/L, HDL-2 1.100–1.125 kg/L, HDL3 1.125–1.175 kg/L, and HDL-4 1.175–1.210 kg/L). The signal integrals GlycA (2.03 ppm) and GlycB (2.07 ppm) arising from glycosylated amino sugars in sidechains of acute phase glycoproteins such as α-1 N-acetyl-glycoprotein were determined from the JEDI-PGPE spectra.

**Data Processing and Statistical Analysis of NMR spectral data**

Following spectral preprocessing and normalization to the eretic signal in *R*, using the “Metabomate v0.5” package, initial Principal Component Analysis (PCA) was carried out to identify extreme outliers and inspect sample variability. Supervised orthogonal partial least square discriminant analysis (OPLS-DA) was performed to identify systematic metabolic differences between the prior burn injury and control groups. Statistical significance and robustness of OPLS-DA models were then validated by permutation testing of the Y matrix for 1000 permutations. The obtained p-values were adjusted using Benjamini-Hochberg (BH) correction^6^ with a false discovery rate (FDR) threshold of 0.05. Statistical TOtal Correlation SpectroscopY (STOCSY) was used to establish structurally correlated signals from the same molecule and pathway correlations between molecules. Briefly, STOCSY calculates a correlation matrix from the apex of each ‘candidate’ marker peak to all other data points in the spectrum, providing a correlation weighting for each peak association^7^.

Weighted-node Metabolite Correlation Network Analysis (WMCNA) was adapted from Weighted Gene Correlation Network Analysis (WGCNA)^8^ and restricted to only significant features. Features of significance were determined using Mann Whitney U testing, corrected for multiple testing (FDR, Benjamini-Hochberg) (*q* value <0.01). Spearman’s correlation was performed to interrogate the relationship between significant lipoproteins, glycoproteins and cytokines, mapped using ggplot2 and corrplot visualisations using *R* language. Features of significance were determined using Mann Whitney U testing, corrected for multiple testing (FDR, Benjamini-Hochberg) (*q* value <0.01). Weighted-node correlation networks were used in conjunction with spearman’s correlations and hierarchical edge bundling^9^, to assess the weighting of the association between significant lipoproteins, small molecules and cytokines and visualised using the *R* package, “edgebundleR”.

**References:**

1. Johnson, B. Z. *et al.* Pediatric Burn Survivors Have Long-Term Immune Dysfunction With Diminished Vaccine Response. *Front Immunol* **11**, 1481 (2020).

2. Dona, A. C. *et al.* Precision high-throughput proton NMR spectroscopy of human urine, serum, and plasma for large-scale metabolic phenotyping. *Anal Chem* **86**, 9887–9894 (2014).

3. Nicholson, J. K., Foxall, P. J. D., Spraul, M., Farrant, R. D. & Lindon, J. C. 750 MHz 1H and 1H-13C NMR spectroscopy of human blood plasma. *Anal Chem* **67**, 793–811 (1995).

4. Nitschke, P. *et al.* J-Edited DIffusional Proton Nuclear Magnetic Resonance Spectroscopic Measurement of Glycoprotein and Supramolecular Phospholipid Biomarkers of Inflammation in Human Serum. *Anal Chem* **94**, 1333–1341 (2022).

5. Jiménez, B. *et al.* Quantitative Lipoprotein Subclass and Low Molecular Weight Metabolite Analysis in Human Serum and Plasma by ^1^H NMR Spectroscopy in a Multilaboratory Trial. *Anal Chem* **90**, 11962–11971 (2018).

6. Benjamini, Y. & Hochberg, Y. Controlling the False Discovery Rate: A Practical and Powerful Approach to Multiple Testing. *Journal of the Royal Statistical Society: Series B (Methodological)* **57**, 289–300 (1995).

7. Cloarec, O. *et al.* Statistical Total Correlation Spectroscopy:  An Exploratory Approach for Latent Biomarker Identification from Metabolic 1H NMR Data Sets. *Anal Chem* **77**, 1282–1289 (2005).

8. Langfelder, P. & Horvath, S. WGCNA: An R package for weighted correlation network analysis. *BMC Bioinformatics* **9**, 1–13 (2008).

9. Barrat, A., Barthélemy, M., Pastor-Satorras, R. & Vespignani, A. The architecture of complex weighted networks. *Proc Natl Acad Sci U S A* **101**, 3747–3752 (2004).
